# Optimization Rules for SARS-CoV-2 M^pro^ Antivirals: Ensemble Docking and Exploration of the Coronavirus Protease Active Site

**DOI:** 10.3390/v12090942

**Published:** 2020-08-26

**Authors:** Shana V. Stoddard, Serena D. Stoddard, Benjamin K. Oelkers, Kennedi Fitts, Kellen Whalum, Kaylah Whalum, Alexander D. Hemphill, Jithin Manikonda, Linda Michelle Martinez, Elizabeth G. Riley, Caroline M. Roof, Nowreen Sarwar, Doni M. Thomas, Emily Ulmer, Felissa E. Wallace, Pankaj Pandey, Sudeshna Roy

**Affiliations:** 1 Department of Chemistry, Rhodes College, 2000 North Parkway, Memphis, TN 38112, USA; sstoddard7607@tuskegee.edu (S.D.S.); oelbk-23@rhodes.edu (B.K.O.); fitke-21@rhodes.edu (K.F.); whakt2-21@rhodes.edu (K.W.); whakt-21@rhodes.edu (K.W.); hemal-23@rhodes.edu (A.D.H.); manve-23@rhodes.edu (J.M.); martinezlinda367@gmail.com (L.M.M.); rileg-22@rhodes.edu (E.G.R.); roocm-21@rhodes.edu (C.M.R.); sarno-23@rhodes.edu (N.S.); donithomas10@gmail.com (D.M.T.); ulmea-22@rhodes.edu (E.U.); emmaleewallac@gmail.com (F.E.W.); 2 College of Veterinary Medicine, Tuskegee University, 201 Frederick D Patterson Dr, Tuskegee, AL 36088, USA; 3 Walnut Hills High School, 3250 Victory Pkwy, Cincinnati, OH 45207, USA; 4 National Center for Natural Products Research, University of Mississippi, University, MS 38677, USA; ppandey@olemiss.edu; 5 Department of BioMolecular Sciences, Schools of Pharmacy, University of Mississippi, University, MS 38677, USA; roy@olemiss.edu

**Keywords:** coronaviruses, molecular docking, inhibitor design, molecular interactions, COVID-19, molecular dynamics, SARS-CoV-2 main protease, SARS-CoV-2 M^pro^

## Abstract

Coronaviruses are viral infections that have a significant ability to impact human health. Coronaviruses have produced two pandemics and one epidemic in the last two decades. The current pandemic has created a worldwide catastrophe threatening the lives of over 15 million as of July 2020. Current research efforts have been focused on producing a vaccine or repurposing current drug compounds to develop a therapeutic. There is, however, a need to study the active site preferences of relevant targets, such as the SARS-CoV-2 main protease (SARS-CoV-2 M^pro^), to determine ways to optimize these drug compounds. The ensemble docking and characterization work described in this article demonstrates the multifaceted features of the SARS-CoV-2 M^pro^ active site, molecular guidelines to improving binding affinity, and ultimately the optimization of drug candidates. A total of 220 compounds were docked into both the 5R7Z and 6LU7 SARS-CoV-2 M^pro^ crystal structures. Several key preferences for strong binding to the four subsites (S1, S1′, S2, and S4) were identified, such as accessing hydrogen binding hotspots, hydrophobic patches, and utilization of primarily aliphatic instead of aromatic substituents. After optimization efforts using the design guidelines developed from the molecular docking studies, the average docking score of the parent compounds was improved by 6.59 −log_10_(Kd) in binding affinity which represents an increase of greater than six orders of magnitude. Using the optimization guidelines, the SARS-CoV-2 M^pro^ inhibitor cinanserin was optimized resulting in an increase in binding affinity of 4.59 −log_10_(Kd) and increased protease inhibitor bioactivity. The results of molecular dynamic (MD) simulation of cinanserin-optimized compounds CM02, CM06, and CM07 revealed that CM02 and CM06 fit well into the active site of SARS-CoV-2 M^pro^ [Protein Data Bank (PDB) accession number 6LU7] and formed strong and stable interactions with the key residues, Ser-144, His-163, and Glu-166. The enhanced binding affinity produced demonstrates the utility of the design guidelines described. The work described herein will assist scientists in developing potent COVID-19 antivirals.

## 1. Introduction

Coronaviruses cause upper respiratory tract illnesses and can infect multiple species [1]. The first severe coronaviruses to impact humans were the severe acute respiratory syndrome-coronavirus (SARS-CoV), which emerged in 2002 [1,2,3] and the Middle East respiratory syndrome (MERS-CoV), which emerged in 2012 [4]. After the emergence of MERS-CoV, researchers expressed the importance of developing future therapeutic interventions for coronaviruses [5]. The current coronavirus which emerged in 2019 is a result of a new strain of severe acute respiratory syndrome-coronavirus 2 (SARS-CoV-2) [6]. This new strain has resulted in a pandemic, termed COVID-19, and has infected almost 15 million individuals worldwide, upending the way of life of individuals across the globe. This COVID-19 pandemic has resulted in over 600,000 deaths between late 2019 and July 2020 [7]. Currently, there are no antiviral therapeutic options available for COVID-19. Although vaccines are being pursued for COVID-19 as a possible therapeutic route, vaccines can have limited effectiveness when strains continually change. Coronaviruses appear to be on a path of continual reemergence with new strains developing, and, as a result, other therapeutic interventions in addition to vaccines must be pursued.

The coronavirus main proteases are essential for the viral RNA to be processed [8]. Currently over 100 crystal structures are available of the specific COVID-19 main protease (SARS-CoV-2 M^pro^). Drug compounds targeting the main protease are being pursued as a major therapeutic route for the treatment of COVID-19. Many of these crystal structures have a compound from the ZINC database [9] bound in the active site or non-specifically bound to an allosteric site that could be further optimized as a drug candidate. Additionally, several peptide or peptide-like inhibitors are being pursued as potential therapeutic agents against the coronavirus main protease [8,10,11,12,13]. The crystal structures 6LZE, 6M0K, 6WNP, 6LU7, and 7BQYof the main protease demonstrates the molecular interactions between four of these peptide-based inhibitors and SARS-CoV-2 M^pro^. The peptide-based inhibitors reported in Zhang, 2020, however, have yet to be crystallized in the SARS-CoV-2 M^pro^ active site [12]. Many scientists are also attempting to repurpose numerous approved drugs for coronaviruses as well [14,15,16,17,18]. When MERS emerged, this process was also pursued as a strategy to develop therapeutic options [19].

The current inhibitors have been developed through virtual screening and further developed using rational drug design. Some docking studies have been performed on already available drug compounds to rank their potential for development as antiviral agents [20,21]. Studies using molecular docking have been utilized to analyze binding interactions of antiviral compounds targeting; the HIV protease [22,23], topoisomerase II DNA gyrase enzymes [24,25], and anti-cancer compounds which target histone deacetylases [26,27,28] and a range of other diseases can assist in the optimization of molecular interactions for drug design. Studies performed to explore the broad substrate binding preferences that SARS-CoV-2 M^pro^ has would therefore significantly assist in the optimization of any lead drug compounds that are being pursued. Furthermore, exploration of the range of binding interactions that can be utilized to strengthen binding would assist researchers in producing the best broad-spectrum options for the current coronavirus and any potential future coronavirus. To address these needs, in this study an ensemble molecular docking approach was performed to probe molecular considerations that can enhance the binding of potential drug candidates to the SARS-CoV-2 M^pro^. The design guidelines discovered were utilized to develop novel optimized inhibitor compounds and optimize the cinanserin drug compound currently being explored by other researchers [10]. All of our novel optimized inhibitor compounds designed outcompeted the zinc database parent compounds and the cinanserin inhibitor.

## 2. Materials and Methods

### 2.1. Preparation of SARS-CoV-2 M^pro^ Receptors for Docking

Two of the main protease X-ray crystal structures for SARS-CoV-2 M^pro^, PDB IDs 6LU7 [10] and 5R7Z [29], were imported into the molecular visualization program UCSF Chimera [30] from the protein databank and saved as mol2 files. Receptors were prepared according to default parameters in Sybyl-X. Protonation states of all residues were established at pH 7.4 during the receptor preparation. The protomol, which defines hydrophobic, hydrogen bond acceptor, and donor regions in the active site, was generated using the native ligand bound in each of the X-ray respective crystal structures. The flexible Surflex-Dock Geom docking protocol was used for all docking analyses.

### 2.2. Preparation of Ligands for Docking into SARS-CoV-2 M^pro^

A total of 220 inhibitor structures were analyzed in this study. The ligands bound in the 67 SARS-CoV-2 M^pro^ receptors available at the time of the start of the study were pooled into one file to evaluate their initial binding affinity. The PDB IDs of receptors utilized are 6LU7 [10], 5RF7, 5REE, 5REC, 5RET, 5RE9, 5R7Z, 5RF8, 6W63, 5RE8, 5RFP, 5RFB, 5REF, 5REL, 5RFK, 5REX, 5RE7, 5REY, 5RFL, 5R85, 5RFY, 5RFW, 5RFH, 5REG, 5RFS, 5R7Y, 5REO, 5RF0, 5RFI, 5RFD, 5RFR, 5REZ, 5RFX, 5RFQ, 5RF9, 5REW, 5R82, 5REI, 5REV, 5RES, 5RFA, 5RER, 5RE4, 5REJ, 5RED, 5REP, 5REU, 5REK, 5REN, 5RFJ, 5RFT, 5RFV, 5RF5, 5RF4, 5RFO, 5REA, 5REM, 5R80, 5RFZ, 5RFF, 5RE6, 5RFU, 5R81, 5RF3, 5RF6, 5RG0, and 5RF1. Structures were either drawn in PubChem or Chemdraw then converted to the simplified molecular line input entry specification (SMILES) notation. Structures were converted to 3D format in USCF chimera by pasting the SMILES strings into the Build Structure module. Compounds to be docked were combined into several mol2 files for docking. In Sybyl-X the drug-like setting in the ligand structure preparation step was utilized to prepare the parent, derivative, and optimized compounds for docking, except where noted. A conformational sample was also developed in the ligand preparation step for all parent and derivative compounds. For the dataset containing the optimized compounds designed in this work, the option “generate preferred conformer/stereoisomer” was used. A minimum of 20 conformers/stereoisomers were generated for each compound. The most diverse set of conformers/stereoisomers was selected for docking. Additionally, compounds retrieved from crystal structures were also docked in the crystallized conformation.

### 2.3. Docking Ligands into the SARS-CoV-2 M^pro^ Receptor

All compounds were docked into both the 5R7Z and 6LU7 SARS-CoV-2 M^pro^ crystal structures. Binding affinity for all compounds was predicted using the Surflex-Dock Geom (SFXC) protocol for all docking simulations. The Surflex-Dock Geom protocol default parameters were used for all docking runs. Briefly, parameters were set to start docking with an additional four starting conformations to enable 100 rotatable bonds and 20 poses max and minimize compounds both pre-dock and post-docking. The binding affinities were calculated using the C-scoring method in Sybyl-X and are reported as −log_10_(Kd) values. The empirical scoring function used accounts for hydrophobic contact, polar interactions, and entropic fixation cost in translational, torsional, and rotational degrees of freedom [31,32]. Docking scores are outputted in −log_10_(Kd) values. Thus, a docking score of 7 correlates to a binding affinity of 1 × 10^−7^ M. A larger binding score indicates a stronger binding to the protein receptor. Docking poses were visualized in both Sybyl-X and UCSF Chimera programs. Molecular interactions were also evaluated in both programs. All images were produced in UCSF Chimera.

### 2.4. Structural Evaluation of SARS-CoV-2 M^pro^ Receptors

Structural characteristics of the coronavirus main protease protein were evaluated in UCSF chimera. Characterization of the hydrophobicity and electrostatic potential map of SARS-CoV-2 M^pro^ was performed to further understand binding preferences to docked substrates. All analysis and development of hydrophobicity and electrostatic potential were performed in UCSF Chimera.

### 2.5. Conservation Analysis of SARS-CoV-2 M^pro^ Receptor

The degree of conservation of residues in the main protease was determined with the program ConSurf [33,34]. The 6LU7 PDB file was submitted to the ConSurf server. Default parameters were used to calculate conservation results. Each receptor was recolored in Chimera to indicate the degree of conservation using the ConSurf coloring scheme, and then the results were evaluated.

### 2.6. Calculation of Physiochemical Properties and Bioactivity

LogP, LogS, topological polar surface area (TPSA), and bioactivity were predicted for optimized drug candidates. Prediction of LogP and LogS values for compounds was performed using ALOGPS 2.1 [35]. SMILES strings for compounds were uploaded into the online server and submitted for calculation of LogP and LogS values using the non-java interface. The topological polar surface area and the bioactivity of all optimized candidates were calculated using the program Molinspiration [36,37]. SMILES strings were uploaded to the molinspiration server and the Calculate Properties and Predict Bioactivity options were selected.

### 2.7. Molecular Dynamics of CM02, CM06, and CM07 Complex with SARS-CoV-2 M^pro^ Receptor (6LU7)

The top three compounds (CM02, CM06, and CM07) that showed a stronger binding to the protein receptor (larger binding score −log_10_(Kd) values) were subjected to all-atom molecular dynamics (100 ns) using Desmond software, ver. 2.3, 2019.1 (Schrödinger) [38]. The selected ligand-protein complexes were first solvated with a TIP3P [39] explicit water model. Counter ions were added to neutralize the charges. The whole system was neutralized using sodium chloride (NaCl) and set to an ionic strength of 0.15 M. An orthorhombic box was used with a buffer distance of 11 Å for each dimension by the Desmond software. The relaxation protocol was modified as specified in Desmond release 2019 and as described in our previous publication [40]. In brief, five steps were involved in the equilibrium stage. The first step involves Brownian NVT dynamics (constant volume and temperature) with *T* = 10 K and restraints on solute heavy atoms for 1 ns; the second step includes *T* = 100 K, H_2_O barrier, Brownian NPT (constant pressure and temperature), membrane restrained in the Z direction and protein restrained for 100 ps; the third step consists of NPgT, Heating from 100 → 300 K, H_2_O barrier and gradual release of restraints; the fourth step includes NVT production, T = 300 K and no restraints for 500 ps; and the final step involves NPT production, *T* = 300 K, and no restraints for 5 ns. After the equilibration stage, the production run was performed in the NPT ensemble using a Langevin thermostat at a temperature of 300 K and 1.63 bar pressure over 100 ns (time-step 2 fs) with recording intervals of 1.2 ps for energy and 10 ps for trajectory. Simulations were run with the OPLS-2005 force field. Plots and figures were generated with the Desmond simulation interaction diagram (SID) implemented in the Desmond software [38].

## 3. Results and Discussion

### 3.1. The SARS-CoV-2 M^pro^ Active Site

**Figure 1:**
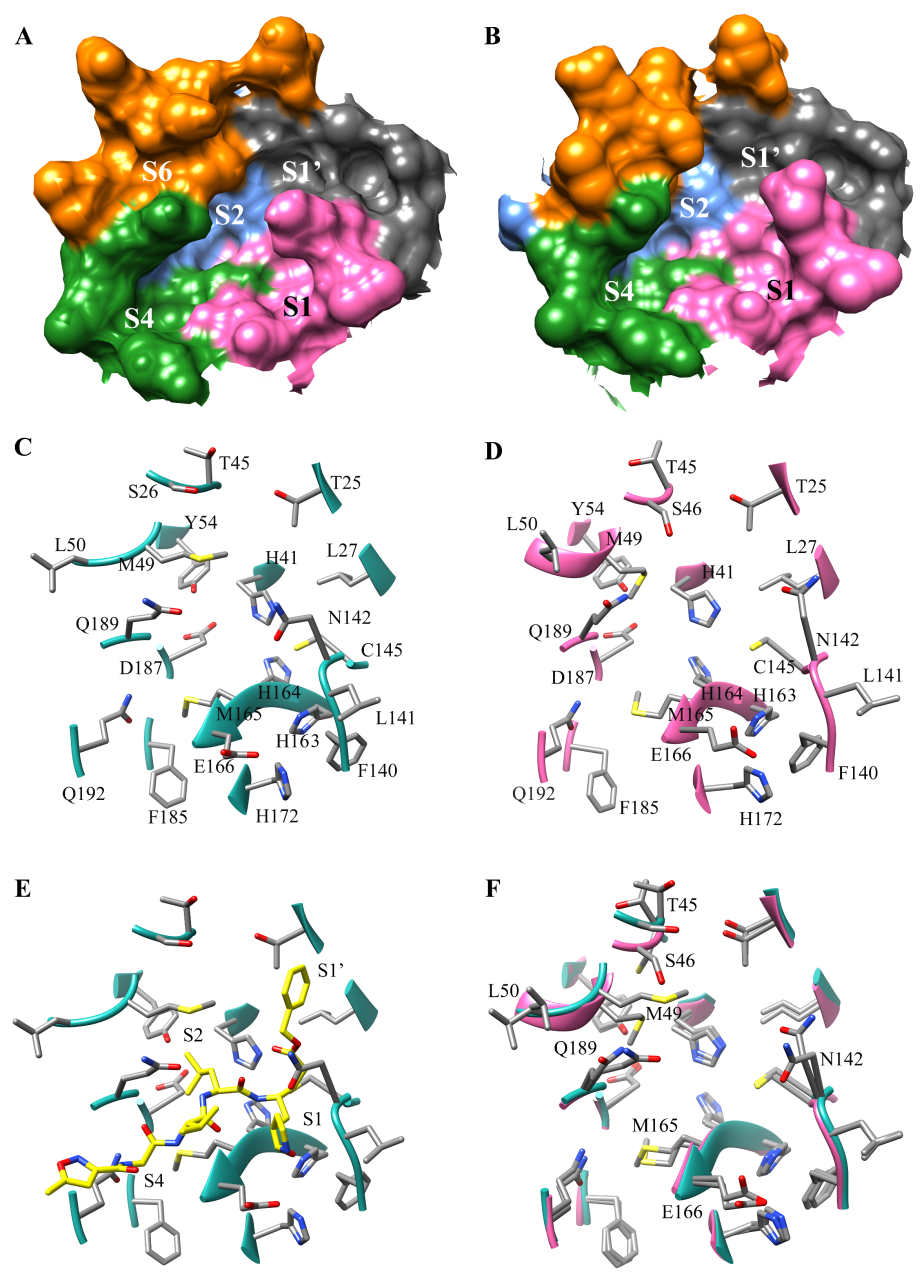
Overview of SARS-CoV-2 Active site using PDB 6LU7 and 5R7Z: (**A**) Surface of SARS-CoV-2 M^pro^ active site in PDB 6LU7, regions colored represent the binding subsites S1, S1′, S2, S4, and the cleft S6, which is accessible in the 6LU7 receptor; (**B**) Surface of SARS-CoV-2 M^pro^ active site in PDB 5R7Z, regions colored represent the binding subsites S1, S1′, S2, S4. (**C**) Residues comprise the key active site regions in SARS-CoV-2 M^pro^ (PDB 6LU7); (**D**) Residues which make of the key active site regions in SARS-CoV-2 M^pro^ (PDB 5R7Z); (**E**) The ribbon structure of the 6LU7 crystal structure of SARS-CoV-2 M^pro^ showing residues contributing to the S1, S2, S4 subsites. The N3 inhibitor is shown to assist in visualizing the subsite binding pockets. (**F**) Overlay of the 6LU7 (cyan) and the 5R7Z (pink) active sites. The highlighted residues are the residues shown to have different side-chain orientations.

Coronaviruses typically have several binding pockets within their protease which makes up the active site. Based on the crystal structure SARS-CoV-2 M^pro^ (PDB ID 6LU7) four binding pockets, S1, S1′, S2, and S4, are present [10]. The side chains of residues Phe-140, Asn-142, Ser-144, Cys145, His-163, His-172, and Glu-166, and the backbone of Leu-141, Gly-143, His-164, and Met-165 create the S1 binding site (Figure 1). The S1′ site is created by the side chains of His-41, Val 42, Asn-119, Thr-25, Cys-145, Gly-143, and the backbone of Thr-26. Subsite S2 is formed by the side chains of Tyr-54, Asp-187, Met-49, and His-41, and the backbone of Arg-188. The last subsite S4 is created by the side chains of Met-165, Leu-167, Pro-168, Ala-191, Gln-192, and the backbones of residues Glu-166, Arg-188, Thr-190. The surfaces and ribbon structure of the actives site for SARS-CoV-2 M^pro^ 6LU7 and 5R7Z crystal structures are shown in Figure 1A–E.

**Figure 2:**
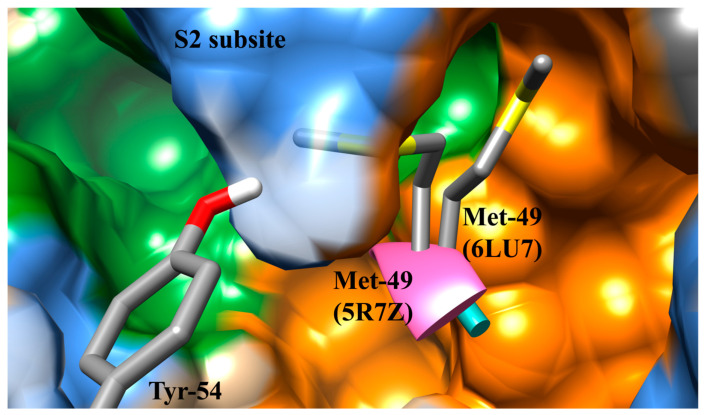
Impact of Met-49 in the length of S2 subsite. The orientation of the residue Met-49 in the 5R7Z receptor decreases the length of the S2 subsite.

Proteins are flexible; thus, the shape of the active site can change as they move. Proteins can be crystallized in multiple conformations resulting in variations of the side-chain orientations in the residues located in a protein’s active site. A comparison of the crystal structures for SARS-CoV-2 M^pro^ 6LU7 and 5R7Z was performed to determine structural variations that could contribute to differences in binding poses. When overlaying 6LU7 and 5R7Z, the crystal structure’s residues Thr-45, Ser-46, Met-49, Leu-50, Asn-142, Met-165, Glu-166, and Gln-189 were observed to have side chains in different orientations (Figure 1F). The most significant variation to the active site can be seen when comparing Met-49. The change in orientation decreases the length of the S2 subsite by at least 3.1 Å. When the side chain of Met-49 is up, as seen in 6LU7, then the S2 active site extends all the way to the hydroxyl group of Tyr-54. When the Met-49 side chain is down then the Tyr-54 becomes buried (Figure 2). A second variation that can be seen when comparing these two receptors is the cleft on the surface that is created behind the S2 pocket in 6LU7, which is significantly smaller in 5R7Z (Figure 1A,B). This cleft represents another accessible site for binding. These observations taken together demonstrate the need for researchers to consider the impact of the multiple conformations on the S2 pocket and binding cleft. The use of multiple conformations when using docking will assist in the prediction of new antivirals agents targeting SARS-CoV-2 M^pro^ as the diversity of accessible variations can produce distinct binding poses for an inhibitor compound. Compounds not docked in multiple receptors may produce false negatives if not considered through a holistic methodology. As we will show in this study, the conformation of Met-49 can contribute to drastic differences in binding poses and affinity.

**Figure 3:**
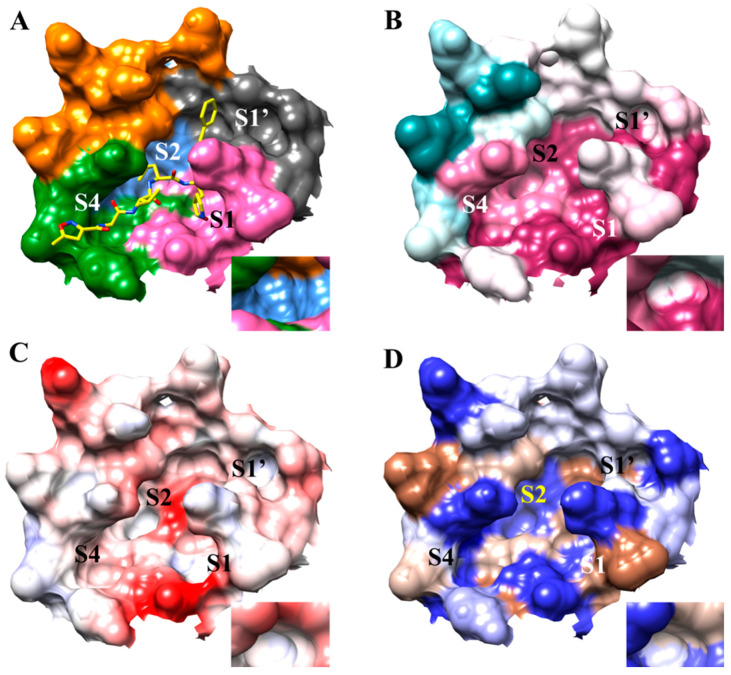
Analysis of binding subsites of SARS-CoV-2 active site using PDB 6LU7: The larger image is the overall active site. The S2 subsite is shown in the smaller image in each panel. This orientation shows the characteristics of inside of the S2 subsite that cannot be seen in the larger image. (**A**) Subsites S1, S1′, S2, and S4. N3 inhibitor is shown for orientation; (**B**) Conservation of subsites. Pink and magenta represent regions that are more conserved, dark cyan and light cyan represent regions that are less conserved. White represents regions without a high or low degree of conservation. (**C**) Electrostatic potential map of subsites. Red represents areas of negative charge density, while blue represents regions of positive charge density. White represents neutral regions. The darker the red or the blue, the greater the degree of negative or positive charge density. (**D**) Hydrophobicity plot of each subsite. The sienna-colored areas represent regions of hydrophobic character. The blue regions represent areas of hydrophilic character. The darker shades of sienna or blue represent greater hydrophobic or hydrophilic character.

The properties of each of the substrate binding pockets were analyzed using conservation analysis, electrostatics, and hydrophobicity to understand the characteristics of each subsite (Figure 3). This information can assist in better rational design of drug compounds. The S1 and S1′ substrate binding sites were determined to be highly conserved, while the S2 and S4 did consist of residues with a lower degree of conservation (Table 1). In the S4 subsite, the residues Thr-190 and Ala-191 were slightly variable in SARS-CoV-2 M^pro^, while Val-193 was highly variable. The residue Met- 49 of the S2 subsite was shown to be slightly variable while the Leu-50 forming the cleft behind the S2 pocket was demonstrated to be highly variable. These variations are important to note as broad-spectrum inhibitors should attempt to bind to very conserved residues. Thus use of the cleft, even though this can represent another binding site, for broad-spectrum design will need to be further studied. It is possible that these variable residues could confer specificity between coronaviruses, therefore inhibitors which can be created to selectively target these residues could be helpful in differentiating between the biological mechanisms utilized between different coronaviruses, if any.

An evaluation of the electrostatics showed the active site has several negatively charged regions. The most electronegative subsites were sites S1′ and S4. Due to the electronegative character of the SARS-CoV-2 M^pro^ active site, positively charge substituents represent a viable option to increase binding affinity, as will be demonstrated in this study. SARS-CoV-2 M^pro^ also has several hydrophobic regions located in each subsite and in the cleft behind subsite S2. These hydrophobic regions permit hydrophobic contacts in the active site and this study will also demonstrate the importance of accessing these patches to increase binding affinity. Further implications of these structural insights will be discussed as the docking studies are explored.

### 3.2. Docking of Bound Zinc Database Inhibitors in Coronavirus Receptors

**Figure 4:**
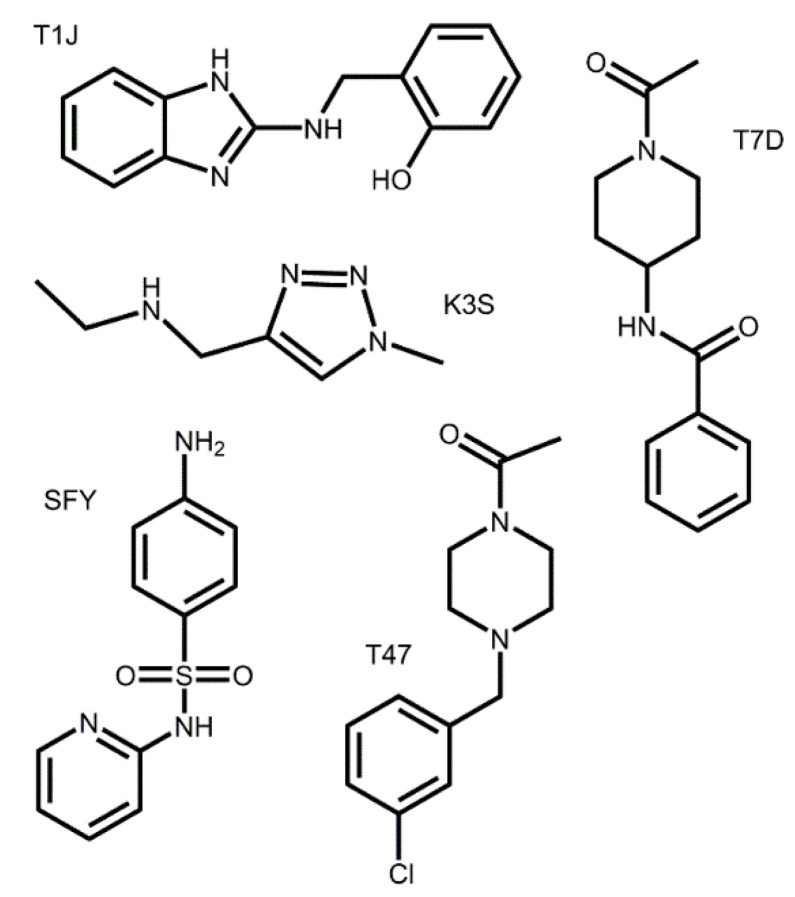
Structures of the five zinc database inhibitors selected as parent structures for further docking analysis.

Due to the variation, both receptors were used to evaluate the docking of all compounds. The highest binding score was accepted as the preferred mode of binding and further analyzed. The docking scores for the zinc database inhibitors that were successfully docked are listed in Supplementary Table S1. Five of the docked zinc database inhibitors, T1J, SFY, T47, K3S, and T7D (Figure 4 and Table 2), produced docking scores of 5.92, 5.42, 4.69, 3.80, and 3.47, respectively. These inhibitors were selected as templates to investigate the impact of structural variations of inhibitor compounds on the SARS-CoV-2 M^pro^ receptor. In the crystal structures containing T1J, SFY and K3S are shown to bind to an allosteric site. The compounds T47 and T7D are shown to be covalently bound to the active site serine in their crystal structures. For the purposes of evaluating binding preferences in SARS-CoV-2 M^pro^, all compounds were docked into the active site of SARS-CoV-2 M^pro^ and used as template reference structures.

### 3.3. Halogens in the SARS-CoV-2 M^pro^ Actives Site Have Little Impact on Binding Affinity

**Figure 5:**
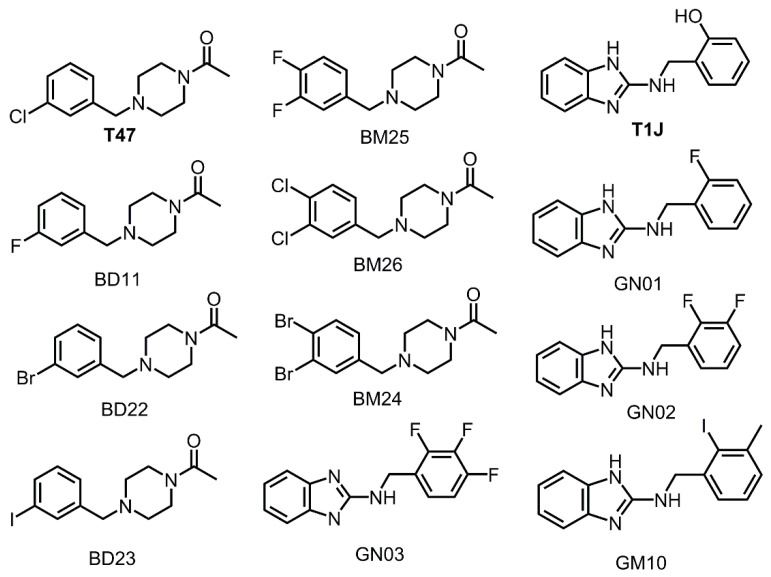
Structures of T47and T1J compound derivatives designed for the halogen dataset.

It was observed that the compound T47 has a Cl atom on the benzene ring, which is located at the S2 subsite of the crystal structure. The majority of the inhibitors published this year as new antiviral options for COVID-19 have not incorporated halogens in the inhibitor structure [9,10,11]. In order to explore the impact of the presence of halogens on inhibitor compounds designed to target SARS-CoV-2 M^pro^, ten compounds were created using T47 and T1J as templates (Figure 5). The impacts of F, Cl, Br, and I in addition to the impact of one, two, or three halogens were explored. The typical mode of binding for the halogenated compounds produced a pose with the halogen atoms in the S2 subsite or the opening of the active site gorge between the S2 or S1′ subsites. The iodine atom of GM10 was shown to site in the S1′ subsite. The T47 compound placed the Cl atom in the S4 subsite. No halogens were placed in the S1 subsite. The docking scores for the parent compounds and the derivatives are shown in Table 3. All ten of the derivative compounds produced docking scores that were similar or lower than the parent compound. These data suggest that halogens do not have a significant impact on binding affinity to the SARS-CoV-2 M^pro^ receptor.

### 3.4. Addition of Aliphatic Substituents Increases Binding Affinity to SARS-CoV-2 M^pro^ Receptor

**Figure 6:**
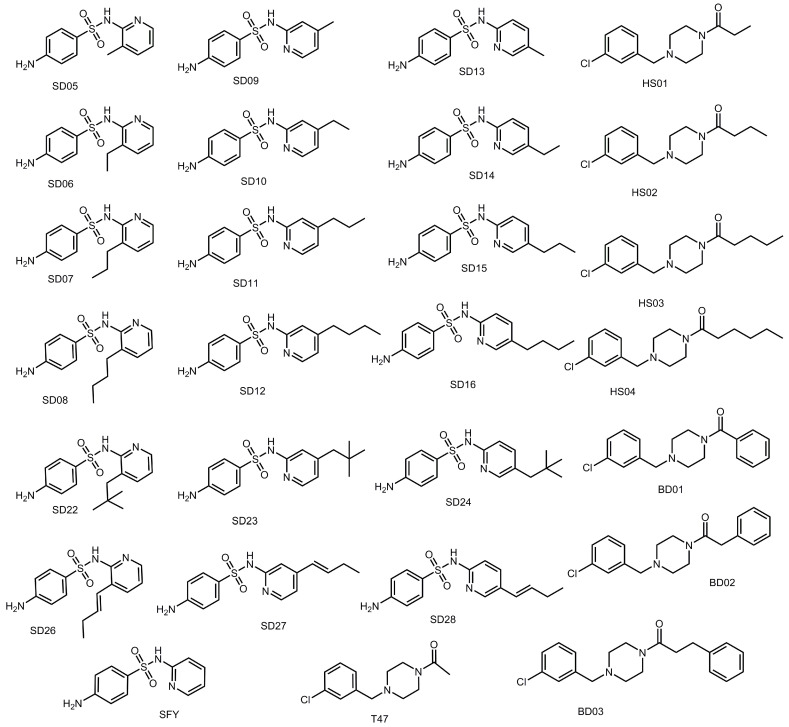
Structures of T47 and SFY compound derivatives designed for aliphatic substituent dataset.

The SARS-CoV-2 M^pro^ active site has several hydrophobic regions. To determine optimal binding interactions using hydrophobic substituents, derivatives of both SFY and T47 were designed by adding hydrophobic substituents to each compound. The impact of the length of the aliphatic substituent on binding affinity was evaluated by adding a methyl, ethyl, propyl, or butyl substituent to SFY and T47. Tert-pentyl substituents and 1-butenyl groups were also used as substituents for SFY derivatives to evaluate the impact of bulky groups and more rigid alkyl groups. For SFY derivatives, the impact of the substituent location was also evaluated by creating ortho, meta, and para versions of each compound. In the T47 derivatives series, the impact of adding a benzene ring and the methylene linkers between the benzene ring and the amide was also interrogated. Collectively, a total of 25 derivatives (Figure 6) were designed, successfully docked, and evaluated.

Docking scores for each derivative are shown in Table 4. All 25 derivatives were demonstrated to produce higher binding affinities than the parent compounds. Compounds HS04, SD12, SD11, SD23, SD15, and SD27 produced docking scores at least 1.5 orders of magnitude greater than the parent compounds. Compounds BD03, SD28, SD08, SD07, and SD24 produced docking scores at least one order of magnitude greater than the parent compounds. The average binding scores for compounds having butyl, propyl, ethyl, and methyl substituents were 6.7, 6.7, 5.7, and 5.6 −log_10_(Kd), respectively. This trend was also consistent with the derivative compounds DB03, DB02, and BD01, which produced binding scores of 5.92, 5.41, and 5.40 −log_10_(Kd). BD03, which has the most methylene linkers between the benzene ring and the carbonyl carbon, scored 0.51 and 0.52 −log_10_(Kd) better than BD02 and BD01, which had one linker or no linker, respectively. When comparing docking scores of the derivatives against the parent compounds it was clearly demonstrated that increasing the length of the aliphatic substituent results in increased binding affinity. Average binding scores for the meta, para, and ortho compounds were 6.86, 6.47, and 6.10 −log_10_(Kd), respectively. Additionally, the data demonstrated a preference for meta conformers over para and ortho conformers.

**Figure 7:**
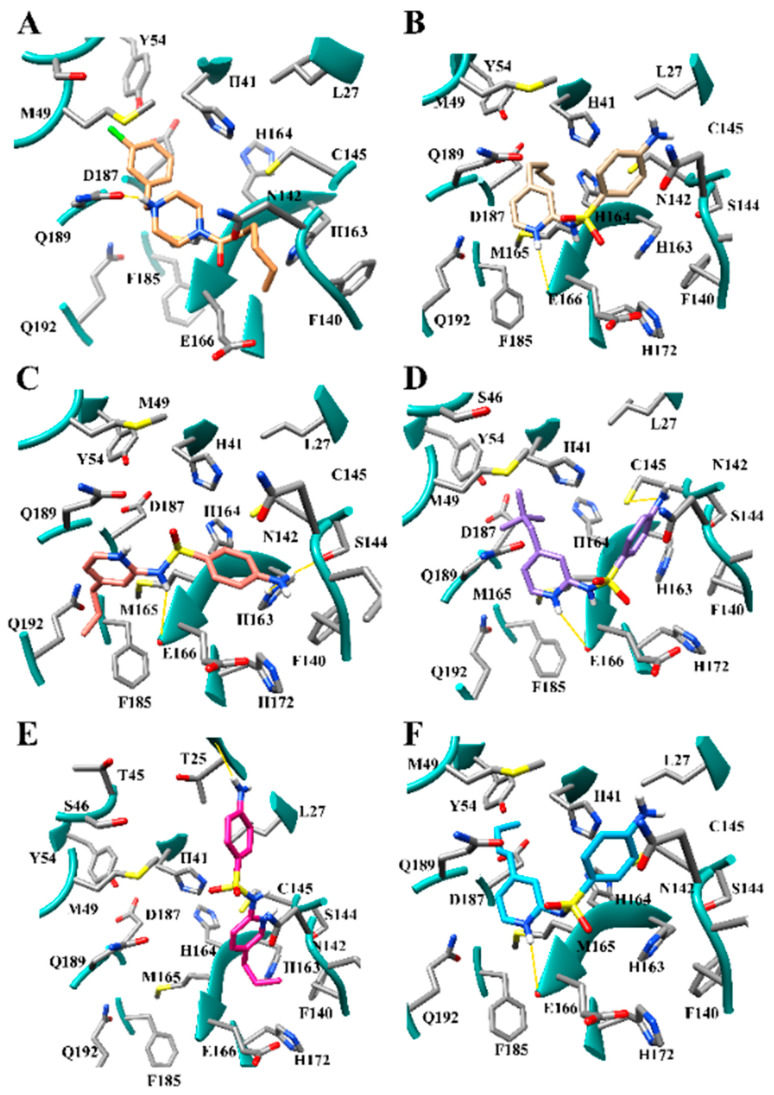
Modes of binding for (**A**) HS04 (sandy); (**B**) SD11 (tan); (**C**) SD12 (salmon); (**D**) SD15 (plum); (**E**) SD23 (deep pink); (**F**) and SD27 (blue) compounds designed for aliphatic substituent dataset in PDB 6LU7. Hydrogen bonding is indicated by solid yellow lines.

The modes of binding were evaluated further to understand the preference for longer aliphatic substituents and meta substituents. HS04, BD03, HS03, BD02, and BD01 all produced modes of binding which interacted with the S1 and the S2 subsites. HS04, HS03, and BD01 positioned the butyl substituent in the S1 pocket and the chlorinated benzene ring in the S2 subsite. This trend was shown to be reversed in BD03 and BD02 which placed the chlorinated benzene ring in the S1 subsite and the unsubstituted benzene ring in the S2 subsite. HS04 (Figure 7A) was shown to produce a hydrogen bond with Gln-189 and the piperazine ring while HS03 formed a hydrogen bond with the nitrogen in the piperazine ring and backbone of His-164. No hydrogen bonds were produced between HS01, BD03, BD02, or BD01 and the active site. The modes of binding for HS02 and HS01 were significantly different than the modes of binding for the other derivatives in this dataset. HS02 was shown to interact with the S2 and the S4 subsite forming a hydrogen bond with Gln-189. The chlorinated benzene ring was bound in S2 while the ethyl group was positioned in the S4 subsite. HS01 positioned the chlorinated benzene ring in the S1′ subsite and the piperazine ring in the S1 subsite. The best binding pose for all T47 derivatives had piperazine rings that had the nitrogen not involved in the amide bond protonated, which result in a positively charged core on the inhibitor compound. The active site gorge between the S2 and S1′ subsites is electronegative, therefore the positively charged ring would increase the attraction between the inhibitors and the receptor.

Several modes of binding were observed for the SFY derivatives in the active site (Figure 7). The top five binders were SD12, SD11, SD23, SD15, and SD27 (Figure 7B–F). Four of these compounds (SD12, SD11, SD23, and SD27) are meta-substituted, while SD15 is para-substituted. The mode of binding for meta-substituted compounds SD12, SD23, SD27 shows interactions with the S2 and the S1 subsites. S23 has a hydrogen bond to the backbone of Glu-166, the alkyl substituent is bound in the S2 subsite, and the aniline ring is in the S1 subsite. The para-substituted compounds SD28 and SD13 also interact with the S2 and S1 subsites in a similar fashion as the meta-substituted compounds. SD11 and SD09, however, bind differently, positioning the alkyl group in S4 and the aniline ring in the S1 subsite. The increase in binding affinity of SD11 over SD09 is due to the larger alkyl group positioned in the S4 subsite. The para-substituted compounds SD14, SD15, and SD05 bind in a unique mode of binding positioning the alkyl group in the S1 subsite, and the aniline ring is bound in S1′ subsite. SD06, which also binds to the S1 and S1′, binds in the reverse manner, positioning the aniline ring in the S1 and the alkyl substituent in the S1′subsite. The para-substituted compounds SD24 and SD16 interacted with the SARS-CoV-2 M^pro^ active sites in a similar fashion as the SD11. Namely, binding to the S4 and the S1 subsites by positioning the alkyl substituent in S4 and the aniline ring in S1. The ortho-substituted compounds SD08, SD22, SD26 also interact primarily with S4 and S1. SD22 was demonstrated to have a reversed orientation compared to SD08 and SD26, placing the aniline ring in S4 and the alkyl group in S1. Ortho-substituted compounds SD08 and SD26 position the alkyl groups near the S2 subsite, but do not seem to achieve the same depth of penetration that the meta-substituted compounds access. SD07 does penetrate significantly into the S2 subsite. This compound is bound such that it π-π stacks with His-41. This stacking interaction rotates the compound such that the alkyl group is inserted directly into the S2 subsite, resulting in the slightly higher binding affinity for the propyl substituted SD07 over the butyl substituted SD08. SD13 also creates a π-π stacking interaction with His-41.

These results taken together suggest that the S2 subsite plays a significant role in establishing binding affinity with hydrophobic groups. While the S4 subsite is also a major hydrophobic area, groups that could produce hydrophobic interactions in the S2 subsite yield more binding affinity than do groups that position the hydrophobic groups in the S4 subsite. The hydrophobicity plot indicates that the portion of the S2 subsite which is formed by Met-49 is quite hydrophobic. Consequently, inhibitors that could access this hydrophobic patch adjacent to Met-49 in the S2 subsite would have increased binding affinities.

**Figure 8:**
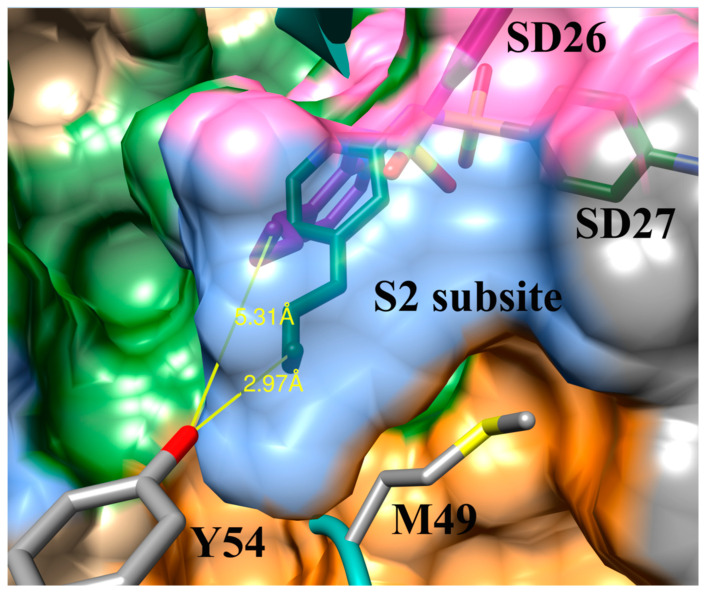
SD26 (purple) and SD27 (green) penetration into the S2 subsite (blue surface) SARS-CoV-2 M^pro^ active site. Depth of penetration by SD27 is 3.22Å deeper than SD26.

The optimal bond angles are important to note as the angling of the ortho-substituted compounds typically did not produce gains in binding affinity that were as significant as the meta- or para-substituted compounds. The para orientation promoted binding to compounds to the active site in a linear conformation, while the meta substitution pattern promotes binding to S2 and S1 subsites efficiently. The ortho conformation decreased the ability of these compounds to access the S2 subsite with the alkyl groups, resulting in weaker binding affinities compared to the meta- and para-derivative compounds. This decrease in S2 subsite access can be explained in terms of the depth of penetration of the alkyl substituent into the S2 subsite. Distances were measured from the Tyr-54 oxygen atom and the methyl group of the butenyl substituent of compounds SD27 and SD26. When comparing differences in depth of penetration into the S2 subsite, the meta-substituted SD27 is 2.97 Å from Tyr-54 while the ortho-substituted SD26 is a distance of 5.31 Å from Tyr-54 (Figure 8). This is a difference of 3.22 Å in depth of penetration into the S2 subsite, increasing SD27′s binding affinity of 7.04 −log_10_(Kd) over SD26′s binding affinity of 6.10 −log_10_(Kd) by almost one order of magnitude. Thus alkyl substituents in the S2 subsite improve binding affinity the most when they penetrate deep into the S2 subsite. The N3 inhibitor which has a leucine residue that sits in the S2 subsite [9] is at a distance of 4.11 Å from the Tyr-54 (measured in the 6LU7 PDB crystal structure). Thus, this inhibitor could be further optimized if the residue was changed to a hydrophobic substituent which could penetrate deeper into the S2 subsite. While the angling of the ortho-substituted compounds decreases the ability of these compounds to access the S2 subsite with the alkyl groups, if these compounds are able to participate in π-π stacking interactions with His-41 they can be successfully directed into the S2 subsite which can serve to direct the alkyl groups into the S2 subsite.

### 3.5. Nitrogen Heterocycles can Increase Binding Affinity to SARS-CoV-2 Active Site

**Figure 9:**
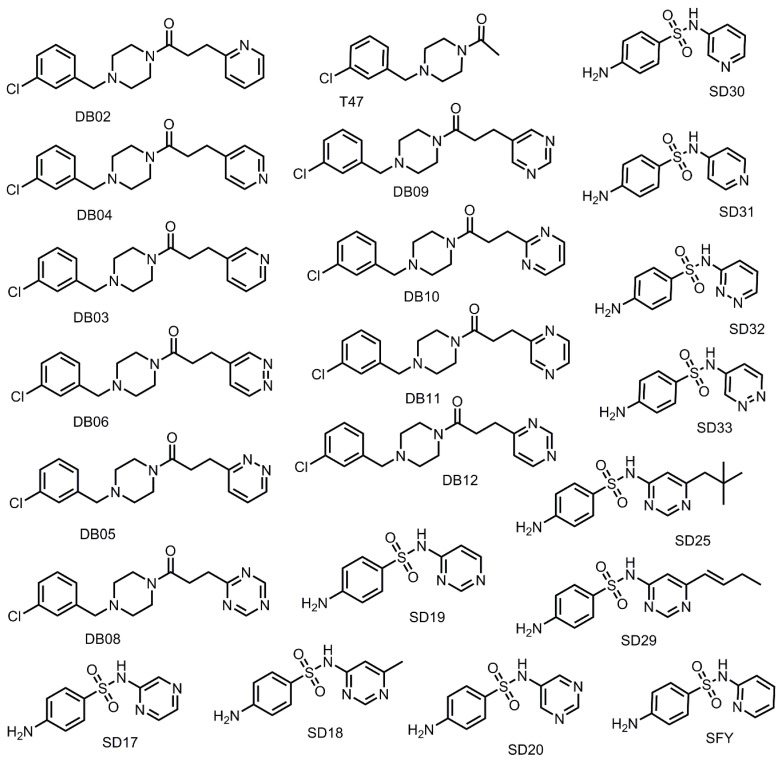
Structures of T47 and SFY compound derivatives designed for nitrogen heterocycle dataset.

Nitrogen atoms permit increased hydrogen bonding interactions and can have different effects on π-π stacking interactions in the active site [41]. Thus, in this study, we substituted the benzene rings with nitrogen-containing heterocycles to determine what impact inclusion of these heteroatoms would have on binding affinity. Twenty derivates of SFY and T47 were designed and successfully docked into the SARS-CoV-2 M^pro^ active site (Figure 9). The number of nitrogen atoms and the location of the nitrogen atoms in the ring structure were evaluated by creating derivatives which contained either pyridine, pyrimidine, pyridazine, or triazine rings. The docking scores for these compounds are listed in Table 5.

**Figure 10:**
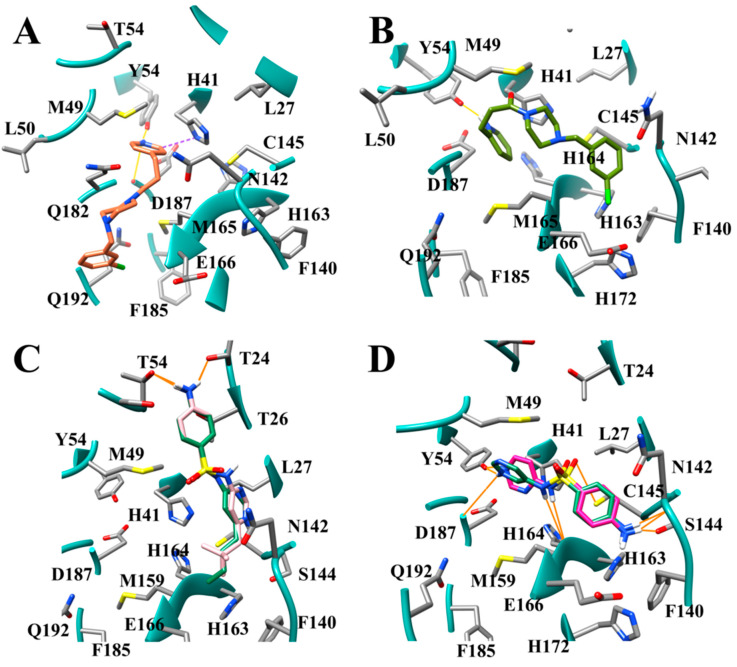
Modes of binding of nitrogen-containing heterocycles. (**A**) DB04 (coral); (**B**) DB02 (olive); (**C**) SD29 (green) and SD25 (pink); (**D**) SD19 (hot pink) and SD20 (sea green). Hydrogen bonds are indicated by solid yellow or orange lines, π-π stacking interactions are shown in purple dash lines.

For the T47 derivatives, inclusion of a second nitrogen produced increases in binding affinity to SARS-CoV-2 M^pro^. Comparing compound BD03, which has no nitrogens atoms in the aromatic ring, to compounds DB04, DB03, and DB02, increases of 0.96, 0.73 and 0.4 were observed, respectively. Both DB04 and DB02 produced a hydrogen bond to Tyr-54, placing the nitrogen-containing pyridine ring in the S2 subsite. However, the pyridine ring of DB04, which has the nitrogen atom in the para position, is able to hydrogen bond by penetrating straight into the S2 subsite. Because DB04 is able to penetrate straight into the S2 subsite, it permits the formation of a hydrogen bond with the backbone of Asp-187 in addition to being involved in a slipped stack π-π stacking with His-41. The pyridine ring of DB02 has the nitrogen in the ortho position, thus to hydrogen bond to Tyr-54 it must bend the methylene linkers to position the nitrogen atom near the hydroxyl group on Tyr-54 (Figure 10A,B). The bending of DB02 to facilitate hydrogen bonding to Tyr-54 results in the loss of ability to hydrogen bond to Asp-187 and produces a weaker edge-to-edge π-π stacking interaction with His-41, subsequently decreasing its binding affinity compared to DB04. DB12 has a pyrimidine ring where one of the nitrogen atoms is in the para position yet produces a binding affinity similar to DB04. While DB12 also produces a hydrogen bond interaction between the nitrogen in the para position on the pyrimidine ring to Tyr-54, the second nitrogen in the ring is located in the ortho position. This results in this hydrophilic nitrogen being positioned against the hydrophobic Met-49 residue. DB04 has a carbon atom in this position on the ring and thus can participate in hydrophobic interactions with Met-49. The reduction in binding affinity between DB12 and DB04 is a result of the more hydrophilic nitrogen interacting with the hydrophobic Met-59 compared to having a more hydrophobic carbon atom of DB04. DB05 and DB06, which produced only small increases in binding affinity over T47, both incorporated pyridazine rings. These pyridazine rings π-π stack against the His-41. In other studies, it has been demonstrated that pyridazine rings are also less favorable when utilized in stacking interactions [26,41], which may be why these compounds did not show significant gains in binding affinity.

SD31, which has a pyridine ring with a nitrogen in the para position, outcompeted the parent compound SFY and SD30. Both SFY and SD30 also contain pyridine rings but have nitrogens in the ortho and meta positions, respectively. The SFY derivatives, having a nitrogen atom in the para positions, showed improvement in binding affinity over SFY, except SD33. All other SFY derivatives (SD20, SD30, SD17, and SD32) produced similar or lower binding scores relative to SFY. Incorporating two nitrogens in the ring showed improvement: strong improvement when comparing SD19 to SFY (increase of 1.44 −log(Kd)), moderate improvement between SD29 to SD27 (increase of 0.68 −log(Kd)), and weak improvement when comparing SD18 to SD09 and SD25 to SD23 (0.11 and 0.02 −log(Kd) increases, respectively). SD19, SD29, SD18, and SD25 all contain pyrimidine rings. SD19 was shown to access hydrogen bonds with Tyr-54 with the para-substituted nitrogen, hydrogen bond with the backbone of His-164 with the sulfonamide amine, and His-163 and Ser-144 with the amine group on the aniline ring. This network of hydrogen bonds aligns the compound in a position to also permit a parallel displaced π-π stacking interaction with His-41. SD29 and SD25 have a different mode of binding compared to the other para-substituted nitrogen compounds (Figure 10C). Both SD25 and SD29 position the aniline ring in the S1′ subsite by hydrogen bonding with Thr-24 and Thr-45. The nitrogen heterocycle is placed in the S1 subsite where hydrogen bonding occurs with the backbone of Cys-145 instead of the S2 subsite hydrogen bonding occurring with Tyr-54. The change in the mode of binding is due to the alkyl substituent on the nitrogen heterocycle. This bulky group would create more steric clashing if the nitrogen compound was positioned to optimize hydrogen bonding interactions with Tyr-54, thus the moiety is placed in the S1 subsite where the alkyl group can spread out and backbone hydrogen bonding can simultaneously be accessed. SD29 and SD25 showed the greatest improvement in binding affinity, which can be attributed to the presence of the butenyl and neopentyl substituents on the ring in addition to the inclusion of a second nitrogen atom in the para position.

The nitrogen atoms placed in the para position of a benzene ring proved to yield the greatest increases in binding affinity. This arrangement allows for optimized hydrogen bonding to Tyr-54 and the backbone of Asp-187 when substituents are not included in the nitrogen heterocycle. The Tyr-54 interaction has, to our knowledge, not been a residue that has been targeted for increased binding affinity by other compounds being developed for COVID-19 [10,11,12]. This work demonstrates a feasible way to access this interaction in the S2 subsite while simultaneously π-π stacking with His-41. While the inclusion of two nitrogens can increase binding affinity, placement is important, as demonstrated by the compounds SD20, SD17, SD32, and SD33 which produced lower binding affinities than the SFY parent compound. Additionally, the azide moiety seems to reduce binding affinity in the active-site gorge. It is notable that SD25 and SD29 produced the best binding scores in this dataset, suggesting that the inclusion of the hydrophobic substituent on the nitrogen heterocycle in addition to para placement of a nitrogen atom would optimize binding interactions to the SARS-CoV-2 M^pro^ active site, albeit through a different mode of binding. The results of this dataset indicate that nitrogen on heterocycles should be at the para position to optimize H-bonding with the hydroxyl group of Tyr-54 in the S2 subsite.

### 3.6. Aliphatic Rings Improve Binding Afinity to SARS-CoV-2 M^pro^ Active Site

**Figure 11:**
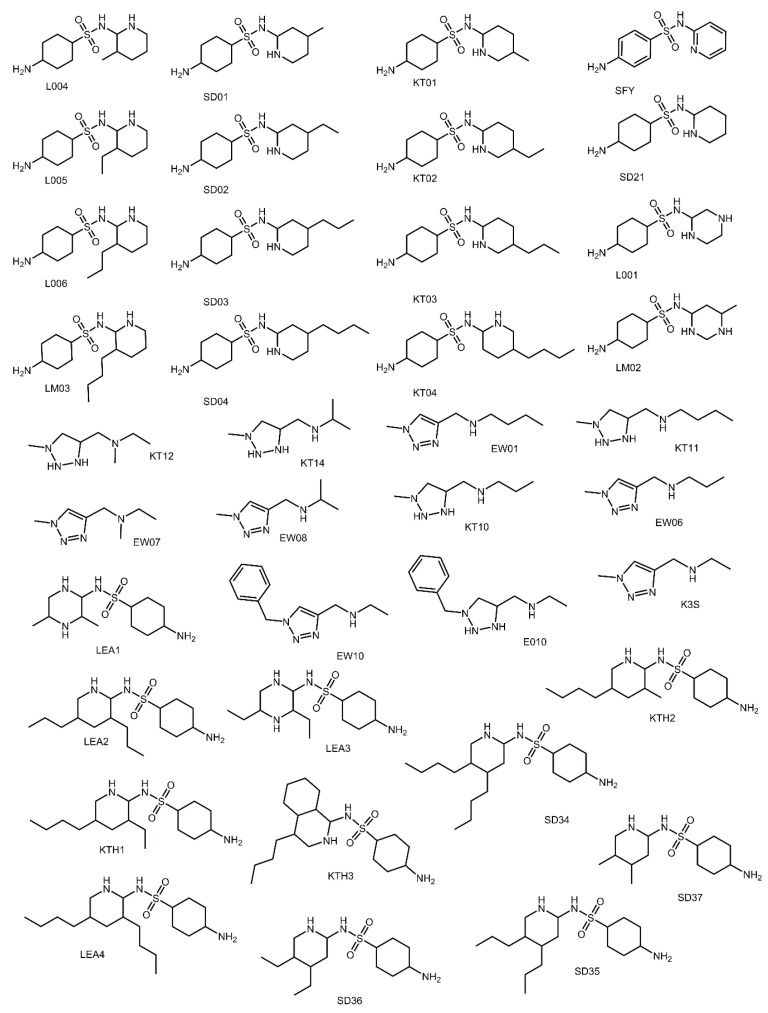
Structures of K3S and SFY compound derivatives designed for aliphatic dataset.

Aromatic versus aliphatic structures can make significant changes in binding affinity to protein receptors. In the design of glioblastoma therapeutics targeting histone deacetylase 4 (HDAC4) inhibitors, it was determined that aliphatic substituents increased the potency of these compounds to the HDAC4 receptor [28]. Thus, in this study the impact of aromaticity versus aliphatic character was also evaluated. Derivatives of SFY and KS3 which were evaluated in the study of aliphatic substituents and the study of nitrogen heterocycles were designed for analysis. The aromatic rings of the previously discussed SFY and KS3 derivatives were substituted with their aliphatic version (i.e., benzene was converted to cyclohexane ring). A total of 36 compounds (Figure 11) were designed and successfully docked. The docking scores for each derivative are shown in Table 6. Using the SFY derivatives, both the impact of substituent length and the impact of nitrogen placement in the ring was evaluated. For the purposes of clarity and easier comparison between structures, the ortho, meta, para nomenclature system which is used for benzene rings was used for the cyclohexane ring instead of the 1,2; 1,3; 1,4 nomenclature system typically used for cyclohexane rings. A new dataset using the KS3 compound was generated, having both aromatic versions and aliphatic versions of the compound. Additionally, the impact of various alkyl group substituents on the nitrogen atoms that were not in the ring structure was determined.

The compounds developed in this dataset had the most significant gains in binding affinity compared to the parent compound. Thirty-five of the 36 compounds produced binding scores greater than the parent compounds. Of those 35 compounds which outcompeted the initial inhibitor, 34 produced docking scores great than 1.5 orders of magnitude or better. Compounds SD34 and LEA4 produced docking scores of 11.02 and 10.94, respectively. This shows an increase of 5.5 orders of magnitude. The docking scores are outputted in −log_10_(Kd) units, thus this unexpected result suggests that these compounds could have as much as 10^5^ times stronger binding affinity against SARS-CoV-2 M^pro^ than the parent compounds. Therefore, incorporation of these features in current antiviral drug candidates being optimized for COVID-19 could yield highly potent inhibitors with low nanomolar inhibition values. Compounds LEA2, KT04, KTH1, KTH3, SD35, KTH2, and LEA3 produced docking scores at least 4.0 orders of magnitude stronger in binding to the SARS-CoV-2 M^pro^ receptor than the SFY parent compound. The ten compounds SD04, SD03, SD36, KT03, LM03, LEA1, L006, KT02, SD37, SD02, KT11, and E010 produced binding affinities at least 3.0 orders of magnitude greater than the parent compound. These seventeen compounds are significantly better than any of the aromatic versions considered in this study.

When considering the twelve monosubstituted compounds, the preferred placement of the alkyl substituent was in the S2 subsite (L004, L006, LM03, SD02, KT03, KT04), then the S4 subsite (SD03, SD04, KT01, KT02) and lastly, the S1 subsite (L005 and SD01). With the exception of L005 and SD01, the amino group was placed in S1 and typically accessed in hydrogen bonds with the backbone of Leu-141 and the side chains of Ser-144 and His-163. The amino group of both L005 and SD01 is bound to the S2 subsite through hydrogen bonding with the backbone of Asp-187. Furthermore, SD01 produced a hydrogen bond with the backbone of Met-49. These results further support the trend that increasing the length of aliphatic substituents increases binding affinity to the SARS-CoV-2 M^pro^ receptor and that the S2 subsite plays a strong role in the binding of hydrophobic substituents.

While the aromatic versions of the SFY compounds preferred the meta, then para, then the ortho form of the inhibitor compound, this trend was not totally observed for the aliphatic versions. The average binding affinity for aliphatic SFY derivatives having substituents located in the para, meta, or ortho position was 8.94, 8.77, and 8.38, respectively. The difference in preference between the meta and para can be explained by difference in the structure of the benzene ring versus the cyclohexane ring. The benzene ring is planar and aromatic which introduces more rigidity into the aromatic SFY derivatives. It was noticed that the para versions of the aromatic SFY derivative compounds are much more linear compared to the meta version, which is due to the aromatic planar benzene ring. The planarity decreases the potential to bind to the S1 and S2 subsites simultaneously. The aliphatic SFY derivatives, however, are not constrained by aromaticity and the para-substituted compounds are able to adopt a conformation allowing them to bind to S1, forming hydrogen bonds and bending to also form hydrophobic contacts in S2.

**Figure 12:**
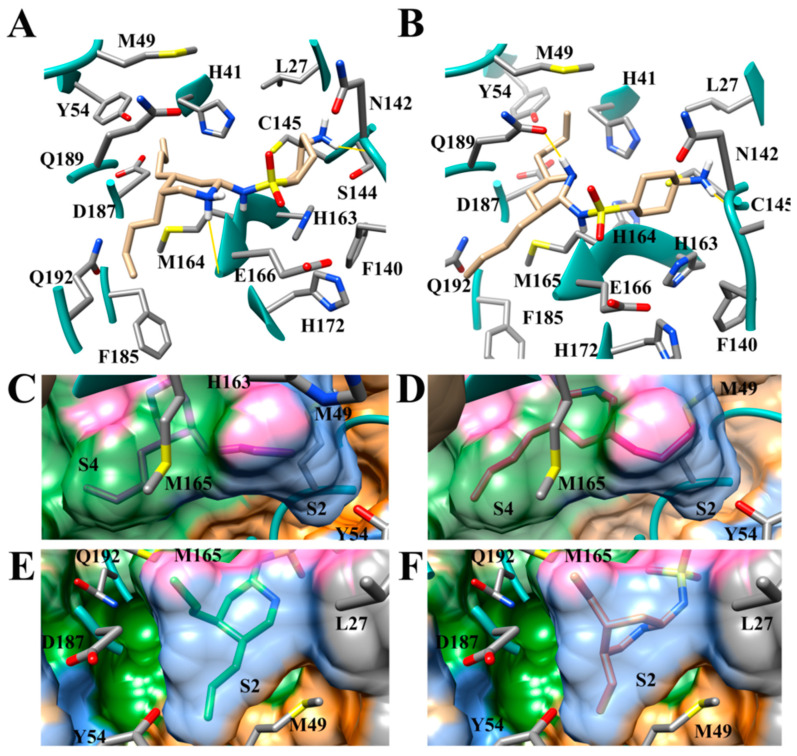
Modes of binding of SFY derivatives. (**A**) SD34 (tan); (**B**) LEA4 (tan); (**C**) SD34 (purple); (**D**) LEA4 (pink); (**E**) SD35 (light green); (**F**) SD36 (brown). In panels (**A**) and (**B**) it can be seen that the aliphatic tails bind in the S2 and the S4 subsites. In panels (**C)** through (**F**) of the surface of the subsites, each of the bound aliphatic substituents is shown. The S2 subsite is colored blue, and the S4 subsite is green. Grey regions are portions of the S1′ subsite. Hydrogen bonds are indicated by solid yellow lines.

After running the single substituted compounds, eleven additional hybrid compounds were designed that had both para- and meta-substituents or substituents located in ortho and para positions (LEA1, LEA2, LEA3, LEA4, KTH1, KTH2, KTH3, SD34, SD35, SD36, SD37). The average docking score for the ortho/para versions was surprisingly larger than the average docking for the meta/para versions, 9.80 compared to 9.65, respectively. The difference in average docking score, however, is not substantial, suggesting that both versions of the compound would be similar in binding affinity. The compounds SD34 and LEA4 produced binding scores of 11.02 and 10.94, respectively, that were significantly better than SFY which only produced a binding affinity of 5.42. These compounds were shown to be able to place one hydrophobic substituent in the S2, the second in S4, and the amine group in S1, accessing all the observed interactions of KT04 and SD04. The binding modes for SD34 and LEA4 are shown in Figure 12a–d. Compounds LEA1, LEA2, LEA3, KTH1, KTH2, and KTH3 were also bound in the same orientation as SD34 and LEA4. However, an unexpected mode of binding was observed with SD35, SD36, and SD37. These three compounds placed both aliphatic tails in the S2 subsite and the amine group in S1. The ability for S2 to accommodate two aliphatic tails demonstrates that large substituents can be placed in this subsite. A key feature that permitted both aliphatic tails being inserted into S2 was that SD35, SD36, and SD37 all had meta/para placements of the substituents. The mode of binding for SD35 and SD36 demonstrating the interaction in the S2 subsite is shown in Figure 12e,f. All other compounds with the exception of SD34 had ortho/para placements of the two substituents, thus these chains were not able to pack closely enough to successfully be inserted into the S2 subsite. Even though SD34 has the ortho/para placements of the substituents, each chain is a butyl group which might limit effective packing of the longer aliphatic chains. The results of this study indicate that inclusion of the aliphatic, not aromatic ring structures will yield SARS-CoV-2 M^pro^ inhibitors with the greatest binding affinity to the active site. Consideration should also be made to fully access the full depth of the S2 subsite.

### 3.7. Several Hydrogen Bonding Hotspots are Present in SARS-CoV-2 M^pro^

**Figure 13:**
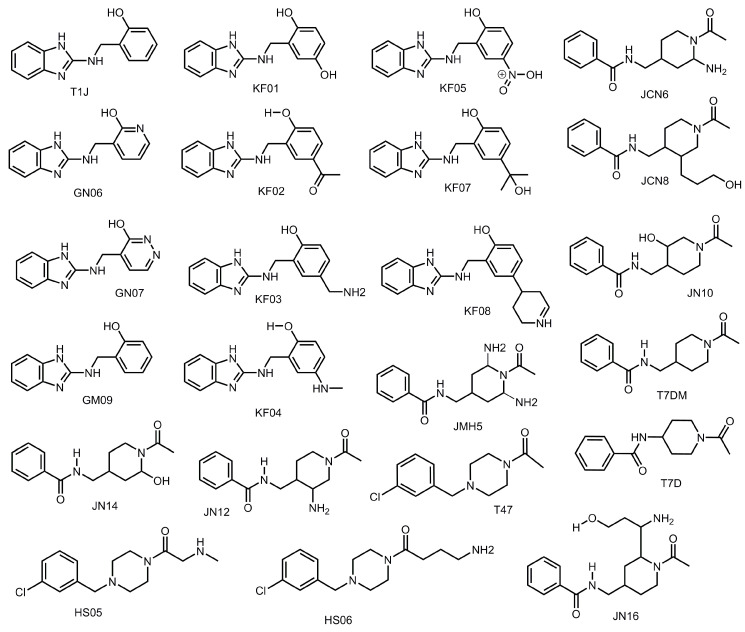
Structures of T7D and T1J compound derivatives designed for hydrogen bonding dataset.

Hydrogen bonding is prevalent in biological systems and can play a significant role in binding interactions. The SARS-CoV-2 M^pro^ has a number of residues in the active site which can hydrogen bond. Thus, regions of the active site which facilitate hydrogen bonding and compound structures that promote access to hydrogen bonding were evaluated. Nineteen derivatives of the scaffolds T1J, T47, and T7D were produced to develop a dataset to interrogate this question (Figure 13). Additionally, six compounds AMM1, AMM3, AMM4, EW11, EW13, and EW14 were created to determine the impact of distance in interacting with multiple hydrogen bonding regions in the active site. The compounds were designed with different variations of hydrogen bond donors and acceptors to identify the regions of SARS-CoV-2 M^pro^ which could best accommodate hydrogen bonding interactions. The docking scores for each compound are listed in Table 7.

**Figure 14:**
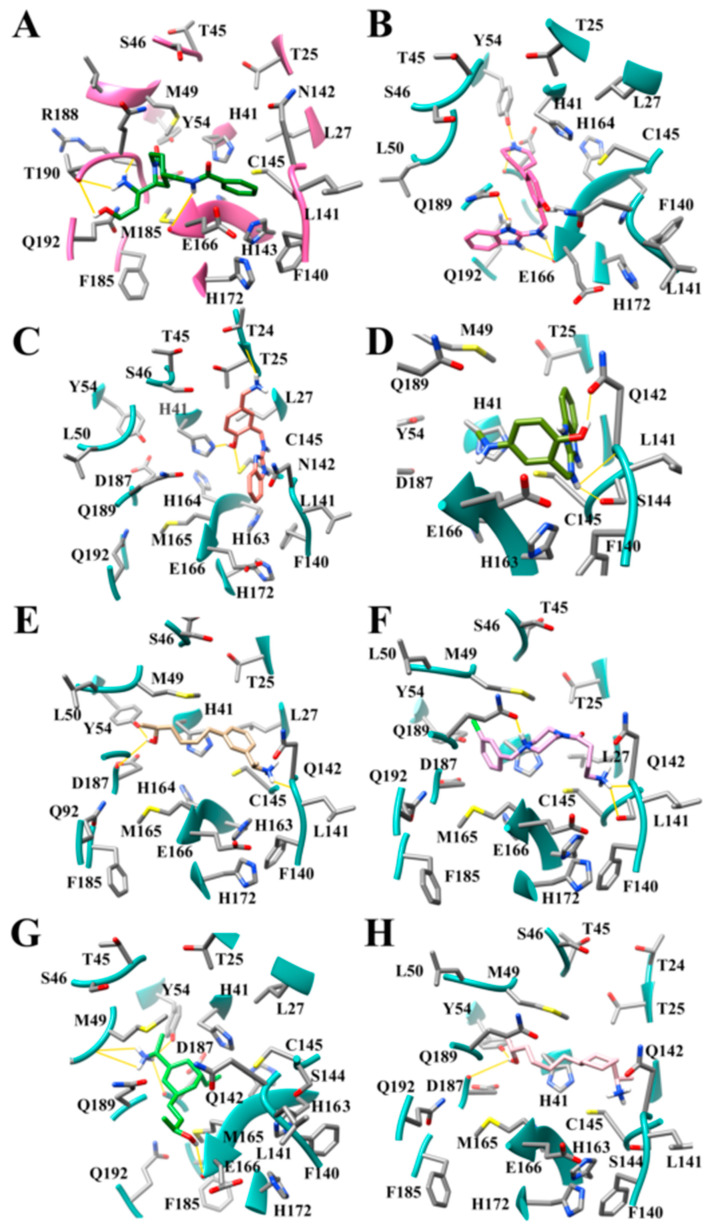
Modes of binding for compounds demonstrating key hydrogen bonding hotspots in the active site of SARS-CoV-2 M^pro^ (**A**) JN16 (green) interacting in the S4 hydrogen bonding hotspot; (**B**) KF08 (pink) hydrogen bonding in hotspots located in the S2 subsite and the active site core; (**C**) KF03 (salmon) hydrogen bonding in hotspots located in the S1′ subsite and the active site core; (**D**) KF04 (olive) hydrogen bonding S1 subsite hotspots; (**E**) EW14 (tan) hydrogen bonding in the hotspots located in the S2 subsite and the S1; (**F**) HS06 (purple) hydrogen bonding hotspots located in the S1 subsite and the active site core; (**G**) AMM2 (lime) shown hydrogen bonding in S2 subsite hotspot and the active site core; and (**H**) AMM4 compounds designed for aliphatic substituent dataset in SARS-CoV-2 M^pro^ receptor. Hydrogen bonds are indicated by solid yellow lines.

In general, inclusion of hydrogen bonding substituents produced improvement in binding affinities for the inhibitor compounds to the SARS-CoV-2 M^pro^. JN16, which was demonstrated to improve the binding affinity of 4.39 orders of magnitude greater than T7DM, was shown to hydrogen bond to the backbone of Arg-188 and Thr-190 by positioning the substituent containing the alcohol and the amine group in the S4 subsite (Figure 14A). The NH in the linker region of GM09 and KF02 also produced hydrogen bonds with Arg-188. The benzimidazole ring nitrogen of GM09, the benzimidazole ring nitrogen, the amine linker of KF05 and KF08, the benzimidazole ring nitrogen, and the alcohol of KF02, the amide NH of JN12 and JN16, and the alcohol of JN14 all produced hydrogen bonds with residue Glu-166. Compounds HS05, HS06, KF08, and JCN8 were shown to produce modes of binding that formed hydrogen bonds with residue Gln-189, across from Glu-166. KF08 produced a mode of binding that allowed for hydrogen bonding interactions spanning in the center of the active site gorge between Gln-189 and Glu-166 (Figure 14B). Hydrogen bonds were formed with histidine rings in the active site; KF03 formed a hydrogen bond to His-41 (Figure 14C), and compounds JN10 and JCN8 formed hydrogen bonds to His-163. Histidine rings, also being aromatic, formed several π-π stacking interactions with compounds in this dataset. His-41 formed stacking interactions with GM09, JCN8, JN14, and JN10, while His-163 formed π-π stacking interactions with JN16. Compound KF05 formed a hydrogen bond with Gln-192 in the S4 subsite. One major area for hydrogen bonding was found in the S1 subsite. In the S1 subsite, the side chains of Ser-144 and Asn-142, in addition to the backbone of residues Leu-141 and Gly-143, were shown to form hydrogen bonds to a number of compounds. The amine group of compound JCN6 was observed to hydrogen bond to Ser-144 and Leu 141; the hydroxyl of JCN8 produced a hydrogen bond to Ser-144; the NH in the linker region of KF04 formed a hydrogen bond with Ser-144 and Leu-141 in addition to forming a hydrogen bond between the hydroxyl and Asn-142 (Figure 14D). The NH in the linker region of JN14 was observed to hydrogen bond with the backbone of residue Gly-143.

Several compounds were evaluated for modes of binding that promoted hydrogen bonding to multiple subsites. When analyzing EW14 it produced a mode of binding which permitted hydrogen bonding to both the S2 subsite and the S1 subsite (Figure 14E). This hydrogen bonding pattern was also seen in several of the compounds in the aliphatic substituent and the aliphatic ring datasets (Figure 10D). HS06 was also able to access hydrogen bonding hotspots in two regions of the active site (Figure 14F). The first was the S1 hotspot and the second was hydrogen bonding in the core which formed a hydrogen bond to Gln-189, and similarly to KF08. Compound AMM2 revealed an additional hydrogen bond that can be accessed in the S2 subsite with the backbone of Met-49 (Figure 14G). This compound was able to produce four hydrogen bonds in the S2 subsite using a simple amine group. The amine group, which in many cases will be protonated at physiological pH, would have three hydrogen atoms. Based on our results, the amine group is an ideal substituent to access the hotspots in each of the subsites. While the amine group seems to be preferred, alcohol substituents are certainly still capable of forming several hydrogen bonds in the S2 subsite (Figure 14H).

When considering the hydrogen bonding patterns of compounds in this dataset and in previous datasets together, five accessible hydrogen bonding hotspots locations can be identified in the SARS-CoV-2 M^pro^ active site. There is one hydrogen bond hotspot in each subsite and in the core of the active site spanning from Gln-189 to Glu-166. The compounds JN12 and JN16 access the hydrogen bonding hotspot identified in the S4 subsite (Figure 14A). Compounds that can access the S4 hydrogen bonding hotspot will successfully hydrogen bond with the backbone of Thr-190 and the backbone of Arg-188 simultaneously. This can be done using either an amino group or a substituent having both an amine and an alcohol separated by a three-carbon linker. The S2 subsite has a hydrogen-bonding hotspot facilitated by Tyr-54, His-41, and the backbone of Asp-187, which can be seen in the mode of binding of the compound KF08 (Figure 14b). We also observed this hydrogen bonding pattern in the previous dataset for the nitrogen-containing heterocycles. KF08 also formed a hydrogen bond pair across the core of the active site between Gln-189 and Glu-166. This represents a third hotspot for hydrogen bonding. The fourth hydrogen bonding hotspot is located in the S1′ subsite near residues Thr-24 and Thr-45 (Figure 10C and Figure 14C). Both the backbone of Thr-24 and the side chains of Thr-24 and Thr-45 can be involved in interacting with the inhibitor compound through hydrogen bonding, creating a forked-like position with the inhibitor in the active site. The S1 pocket has another very prominent hydrogen bonding hotspot created by the side chains of residues Ser-144 and His-163, the backbone of Leu-141 and Gly-143, and sometimes the side chain of Asn-142. While it is possible to simultaneously form a hydrogen bond to all four residues, Ser-144, His-163, Leu-141, and Gly-143, using an amino group (Figure 14D), it was observed that Ser-144 and the backbone of Leu-141 were almost always hydrogen bound together (Figure 14D).

### 3.8. Design of Compounds with Optimized Binding Affinity to SARS-CoV-2 M^pro^

**Figure 15:**
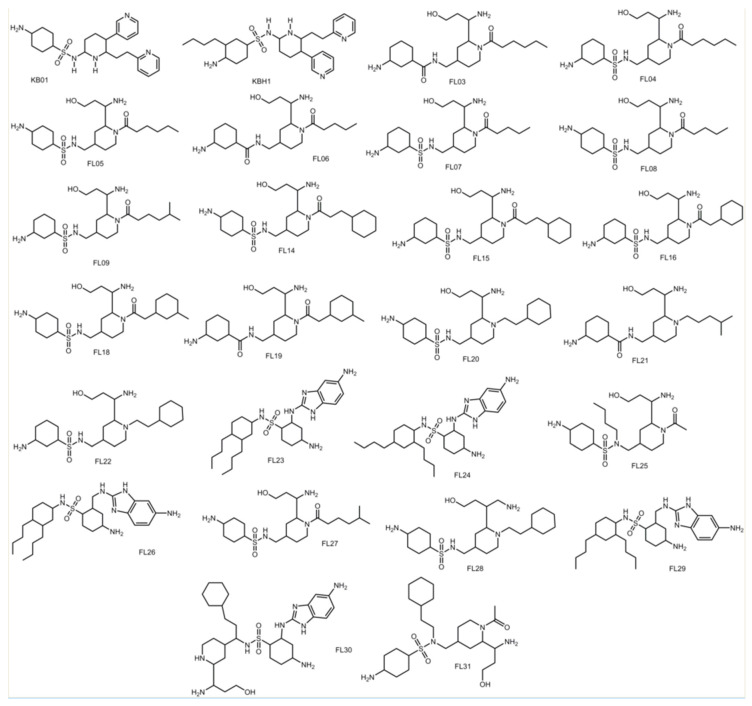
Structures of optimized compounds designed using the guidelines garnered from the molecular docking study.

To determine the overall effectiveness of the guidelines developed in this study, a new dataset was created with twenty-six optimized compounds and given the name Foundations-Lab (FL) compounds series (Figure 15). These compounds were designed and successfully docked into the SARS-CoV-2 receptors. Because the conformational flexibility of the compounds was outside of the Sybyl-X program capabilities, “generate favored Tautomers/Stereoisomers” selection was used for ligand preparation. Five compounds (FL23, FL24, FL26, FL29, and FL30) had molecular weights (MWs) ranging from 519 to 605 Da, which is over the 500 MW cutoff used for Lipinksi’s rules. Thus, for the purposes of evaluating the effectiveness of the rules, the drug-like setting was not used to prepare the compounds in the final hybrid dataset. The docking scores of the FL compounds are listed in Table 8.

**Figure 16:**
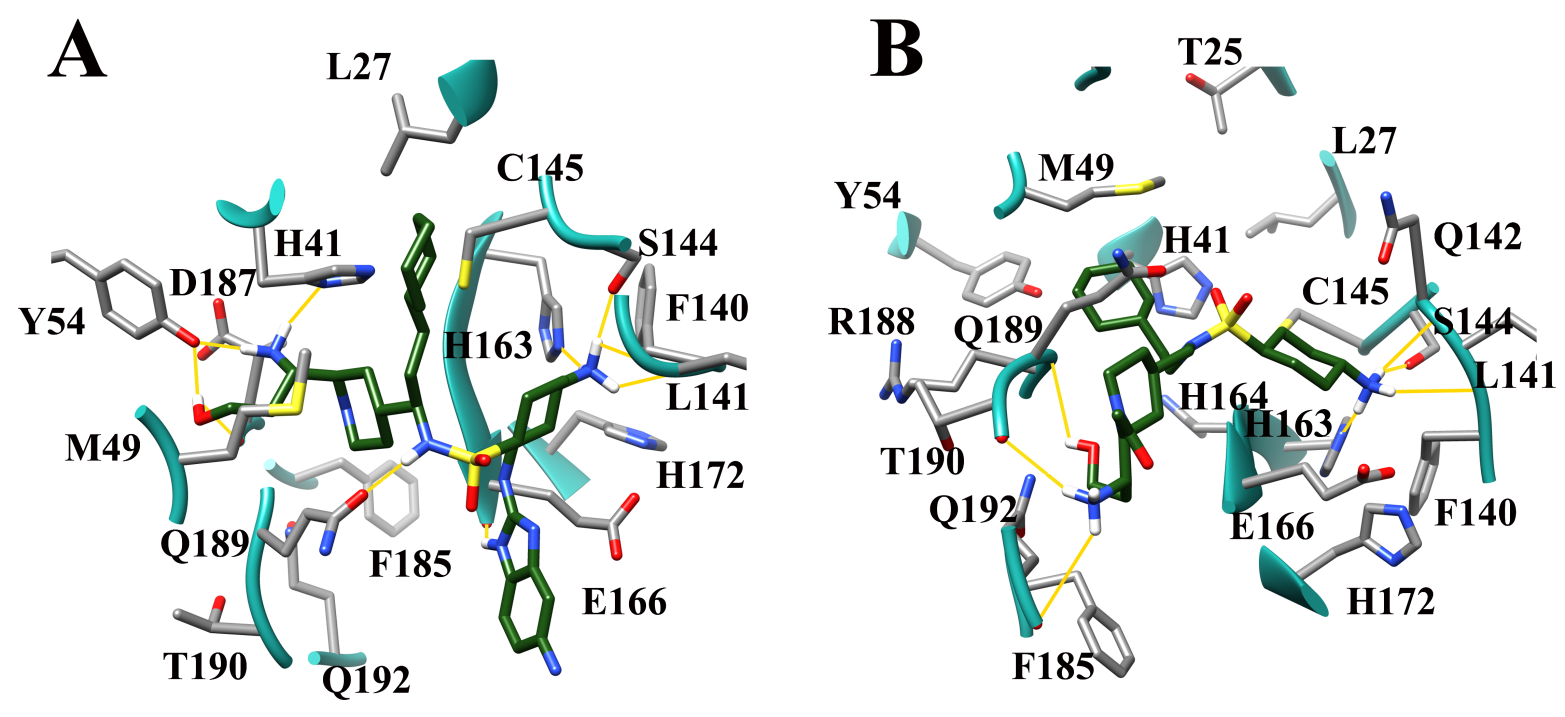
Mode of binding of the top two optimized compounds (**A**) FL30 (green); and (**B**) FL20 (green) designed using molecular guidelines discovered to maximize binding to the SARS-CoV-2 M^pro^ active site.

The FL compounds were produced by selecting the multiple features of the derivative compounds used in the datasets in this molecular docking study. Compounds were designed to strategically interact with multiple hydrogen bonding hotspots in the S1, S1′, S2, S4. Some compounds were optimized to access the S2 subsite with hydrophobic substituents. The FL compounds produced binding scores ranging from 9.26 to 13.98. The average binding scores of the zinc database parent compounds was 4.66 −log_10_(Kd), while the average docking score of the optimized compounds was 11.28 −log_10_(Kd). This represents an overall average increase in binding affinity by 6.62 orders of magnitude. Thus, the FL compounds are predicted to significantly outperform the zinc database parent compounds. These data support the effectiveness of the optimization guidelines developed by the docking experiments. The FL compounds produced modes of binding which access all of the major hydrogen bonding hotspots and the hydrophobic patches (Figure 16), significantly increasing their binding affinity over the parent compounds. FL30 produced a total of ten hydrogen bonds (Figure 16a). The alcohol and amino portion taken from JN16 produced four hydrogen bonds between the amino group and residues Tyr-54, His-41, and Asp-187 in the S2 subsite; the cyclohexane ring with amino substituent portions taking the compounds in the SFY derivatives series formed four hydrogen bonds with residues Ser-144, His-163, Leu-141, of the S1 subsite; and the sulfonamide and benzimidazole ring formed two hydrogen bonds spanning the core of the active site between Gln-189 and Glu-166. The hydrophobic substituent which was intended to interact with the S2 subsite was determined to interact with the hydrophobic patch in the S1′ subsite.

A few of the FL compounds produced modes of binding that were unexpected, yet still accessed the intended molecular interactions albeit with a different substituent than expected. The compound FL20 formed hydrophobic interactions in the S2 subsite, accessed the hydrogen bonding hotspot in the S4 subsite using the alcohol and amino portion taken from JN16, and accessed the S1 hydrogen bonding hotspot (Figure 16b). The molecular features identified in the FL series can be reliably used to design tight-binding inhibitor compounds targeting SARS-CoV-2 M^pro^.

### 3.9. Optimization of Cinanserin Hit for SARS-CoV-2 M^pro^

**Figure 17:**
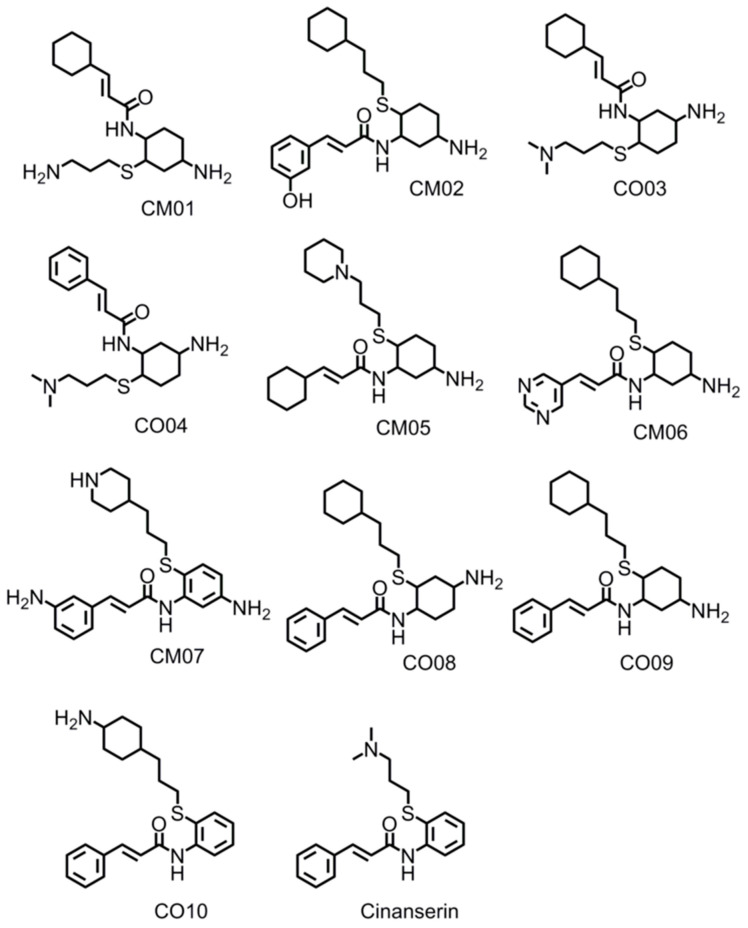
Cinanserin and optimized cinanserin inhibitor compounds.

To further confirm the effectiveness of the selected inhibitors, the compound cinanserin from the work of Jin et al., 2020 [10] was selected for optimization of binding affinity to SARS-CoV-2 M^pro^. Cinanserin has a 125 micromolar IC50 inhibition against the SARS-CoV-2 M^pro^ and produced a docking score of 6.01. Ten compounds were developed using defined guidelines. The docking run was also completed according to the same protocol as the FL inhibitors, wherein the conformational sample was created using the “generate favored Tautomers/Stereoisomers” selection for ligand preparation and drug-like setting disabled. The structures of cinanserin and the optimized inhibitors are shown in Figure 17. The optimized compounds were then docked into SARS-CoV-2 M^pro^ to determine if stronger binding was produced. The docking scores for cinanserin and the optimized compounds are shown in Table 9.

**Figure 18:**
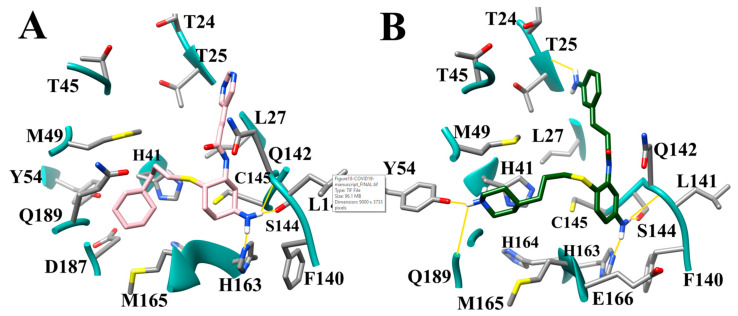
Mode of binding of the optimized cinanserin compounds (**A**) CM06 (pink); and (**B**) CM07 (green).

The docking pose of cinanserin [10] demonstrated that the core benzene ring was placed in the S1 subsite, the terminal benzene was located in the S1′ subsite and the amine group was located in the S2 subsite. Thus, optimization of binding interactions in the S1, S1′, and S2 subsites were prioritized. Briefly, to improve the affinity of cinanserin, aliphatic rings were introduced in some derivatives, hydrogen bonding substituents were strategically placed to access the hotspots in the S1′, S1, and S2 subsites, and larger hydrophobic groups were substituted for the amine group to increase hydrophobic interactions in the S2 subsite. An NH_2_ group was added to the core rings on each of the derivatives to anchor the derivative in the S1 hydrogen bonding subsite. All ten of the optimized compounds outperformed the cinanserin parent inhibitor. The greatest improvement was garnered by CM06 which produced a binding score of 10.60, an increase of 4.59 –log_10_(Kd) over the cinanserin parent compound. The modes of binding produced mirrored the expected binding poses. The modes of binding for the compounds CM06 and CM07 produced the most significant increases in binding affinity and are shown in Figure 18A,B. When considered in conjunction, this demonstrates that current drug compounds that are being pursued as therapeutic options for COVID-19 by targeting SARS-CoV-2 M^Pro^ can be optimized using the molecular guidelines described in this paper and yield significant gains in binding affinity.

The bioactivity of compounds can be predicted to evaluate their potential against certain drug targets. The program Molinspiration predicts the bioactivity of potential compounds to serve as G-protein coupled receptor (GPCR) ligands, ion channel modulators, kinase inhibitors, nuclear receptor ligands, enzyme inhibitors, and protease inhibitors. Because SARS-CoV M^pro^ is a protease, ideally compounds should be predicted to be protease inhibitors. Surprisingly, cinanserin was not predicted to be a protease inhibitor, producing a bioactivity score of −0.10. All but one of the optimized cinanserin compounds, however, were predicted to be protease inhibitors. Compounds CM06, CM05, CM01, and CO03 produced the strongest bioactivity scores for protease inhibitors. No correlation between binding affinity and bioactivity score could be produced as CM01 produced the highest bioactivity score, followed by C003, the CM05, and finally CM06. Based on the data in compound, CM06 might produce an effective low nanomolar SARS-CoV-2 M^pro^ inhibitor. All compounds were predicted to have protease inhibitor activity and had the most significant bioactivity scores. Taken together, this data suggests that not only do the optimization guidelines discovered in this work help to enhance binding affinity to SARS-CoV-2, but they are useful in strengthening the bioactivity of drug candidates to be protease inhibitors overall. The compounds designed in this work could also be used as potential inhibitor compounds for the treatment of COVID-19.

### 3.10. Molecular Dynamics of CM02, CM06, and CM07 for SARS-CoV-2 M^pro^ (PDB ID: 6LU7)

Docking is a static representation of one of the best poses of the molecule in the protein’s active site but does not shed full insight into the binding mode and dynamic hit compounds in the active site. Molecular dynamic (MD) simulations can provide key insight about the atom movements with time and the stability of the protein-ligand complex. The docked pose of the top three compounds (CM02, CM06, and CM07) that showed stronger binding to the 6LU7 (See Table 9) were used for MD studies with the OPLS-2005 force field.

**Figure 19:**
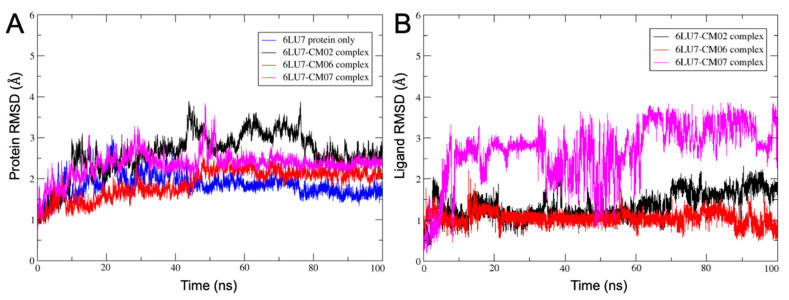
Root mean square deviation (RMSD) analysis of molecular dynamic (MD) simulation trajectory. The RMSD plot obtained for (**A**) **C**-α atoms of the protein SARS-CoV-2 M^pro^ (PDB ID 6LU7) with CM02, CM06 and CM07 complex; and (**B**) ligand-heavy atoms for CM02, CM06 and CM07-SARS-CoV-2 M^pro^ complex (PDB ID: 6LU7), with respect to the reference frame at time 0 ns.

The conformational dynamics and stability of the protein-ligand complexes, CM02, CM06, and CM07, with SARS-CoV-2 M^pro^ (PDB ID:6LU7), were analyzed using the root mean square deviation (RMSD), and by the root mean square fluctuation (RMSF). The RMSD graphs of the protein C-α atoms (Figure 19A) of all three protein-ligand complexes, CM02, CM06, and CM07, indicate that after 10 ns, the proteins have equilibrated, and simulations are converged with respect to the reference frame at time 0 ns. All the frames from 100 ns trajectory were aligned on C-α atoms of the reference frame. In the case of ligand RMSD (Figure 19B), the fluctuation was observed during the first 6 ns of simulation, after that complexes did not undergo any significant conformational changes except for CM07. The RMSF of SARS-CoV-2 M^pro^ (PDB ID:6LU7) was calculated to understand the fluctuation of amino acids interacting with CM02, CM06, and CM07 during the 100 ns simulations. The amino acids of 6LU7 that were involved in interactions with CM02 and CM06 did not show much fluctuation in their RMSF values (Supporting Information, Figure S1), indicating the stability of the biomolecular system. However, amino acids of 6LU7 that were involved in the interactions with CM07 showed significant fluctuations in their RMSF values, indicating a less stable biomolecular system compared to the 6LU7-CM02 and 6LU7-CM06 complex.

**Figure 20:**
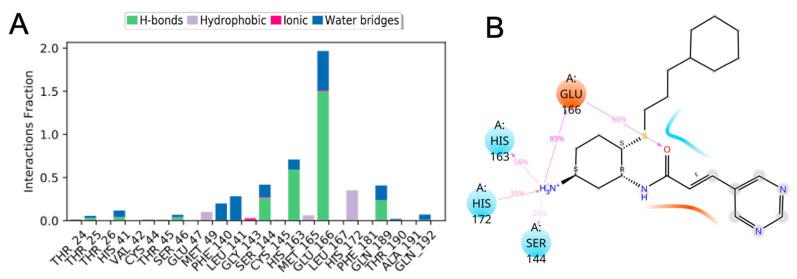
Analysis of (**A**) molecular interactions; and (**B**) type of contacts (2D interaction contour map with the key protein residues) for CM06 with SARS-CoV-2 M^pro^ (PDB ID: 6LU7) after MD simulation. Interactions that lasted more than 10% of the simulation time were considered.

The residue interactions (Figure 20A) and protein-ligand 2D contact contour map (Figure 20B) of SARS-CoV-2 M^pro^ (PDB ID: 6LU7)-CM06 complex shows that Leu-141 (water bridges), Ser-144 (25% contribution (H-bond) and water bridges), His-163 (58% contribution (H-bond) and water bridges), Glu-166 (60% and 89% contributions (side-chain and backbone H-bonds)) and His-172 (35% contribution (hydrophobic)) were crucial amino acids that formed strong and stable interactions with CM06.

**Figure 21:**
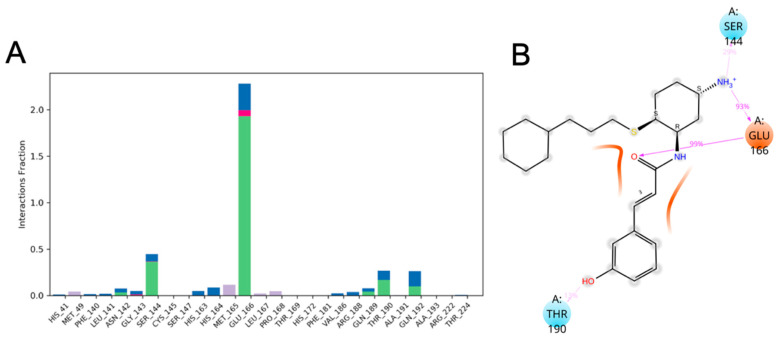
Analysis of (**A**) molecular interactions; and (**B**) type of contacts (2D interaction contour map with the key protein residues) for CM02 with SARS-CoV-2 M^pro^ (PDB ID: 6LU7) after MD simulation.

The interactions histogram (Figure 21A) and protein-ligand 2D contact contour map (Figure 21B) shows the residue interactions of viral protease SARS-CoV-2 M^pro^ (PDB ID: 6LU7) with CM06. These graphs show that Ser-144 (29% contribution (H-bond) and water bridges), Glu-166 (99% and 93% contributions (side-chain and backbone H-bonds), and Thr-190 (13% contribution (H-bond)) were crucial amino acids that formed strong and stable interactions with CM02. Similarly, the interaction histogram and protein-ligand contact contour map of SARS-CoV-2 M^pro^ (PDB ID: 6LU7) with CM07 (Supporting Information, Figure S2) exhibited interactions with Gln-19 (<10% contribution, H-bond) Thr-25 (water bridges), Gln-69 (<10% contribution, H-bond), Asn-119 (<10% contribution, H-bond) and Glu-166 (10% contribution, H-bond, water bridges, and ionic interaction) and identified crucial amino acids that formed interactions with 6LU7. The overall contributions of critical amino acid interactions with CM07 were significantly less during the entire 100 ns of simulation, indicating that this compound diffused slightly with the initial binding pocket of 6LU7 and showed lower affinity towards crucial residues identified for CM02 and CM06.

**Figure 22:**
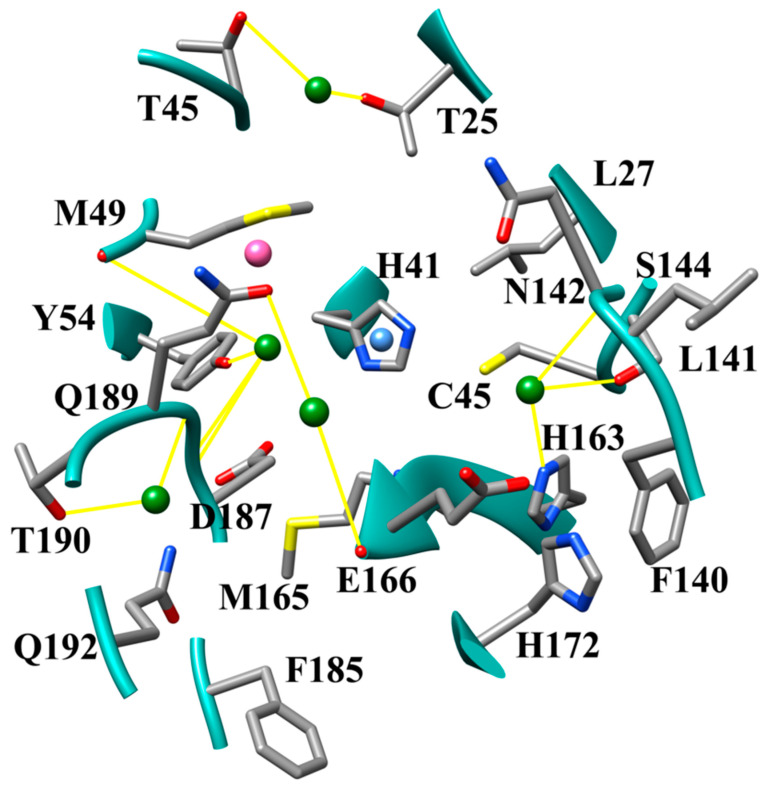
Visual summary of key molecular interactions facilitating strong binding affinity to SARS-CoV-2 M^pro^. Hydrogen bond hotspots are shown as green spheres, with hydrogen bonds shown as yellow lines. The hydrophobic core in S2 pocket is indicated by pink sphere. Blue sphere indicates π-π stacking region.

Taken together, the results of the docking study and the MD simulations demonstrate several key interactions that facilitate improved binding affinity to the SARS-CoV-2 M^pro^ receptor. In Figure 22, a visual summary of the key molecular interactions is shown.

## 4. Conclusions

The ensemble molecular docking study revealed several tools which can be utilized to optimize current drug candidates for SARS-CoV-2 M^pro^ and subsite binding preferences. Binding in the S1′, S2, and S4 subsites can all be facilitated by both hydrophobic interactions or hydrogen bonding interactions. The S2 subsite was one of the most prominent binding sites utilized in the inhibitor mode of binding. Two methods can be successfully employed for drug compounds to access the S2 subsite: the inclusion of hydrophobic substituents that can interact with Met-49, or the inclusion of hydrogen bonding donor or acceptors atoms, which can access the hydrogen bonding hotspot formed by the backbone of residues Asp-187, Arg-188, Gln-189, and the side chain of Tyr-54. Compounds can be developed to access the S1 subsite with high affinity by accessing the hydrogen bonding hotspot formed by Leu-141, Ser-144, His-163, and Asn-142. The most facile way to achieve binding in this region is a protonated nitrogen atom. The hydrogen bonding hotspot in the S1′ subsite can also be easily accessed with an amine group or an alcohol group. Accessing the hydrogen bonding hotspot in the S2 subsite seems to be a molecular guideline that is not currently being employed in the design of COVID-19 inhibitors. Aliphatic ring systems outperform aromatic ring systems and can be an easy change in drug compound structures to produce substantial increases in binding affinity of drug compounds to SARS-CoV-2 M^pro^. However, when using compounds, having an aromatic ring structure inclusion of nitrogen heterocycles or aliphatic substituents can significantly improve binding affinity as long as care is taken to optimize the most effective angle, permitting access to the binding interactions of interest, whether that is hydrophobic interactions or hydrogen bonding interactions in a number of subsites. The optimization guidelines proved to be effective in increasing binding affinity specifically to SARS-CoV-2 M^pro^ and in increasing drug compounds’ potential to be protease inhibitors. The optimized compounds produced in this work used to test the design guidelines and the optimized cinanserin compounds are predicted to be viable drug candidates for COVID-19 therapeutics. MD simulations of optimized cinanserin derivatives CM02, CM06, and CM07 revealed that CM02 and CM06 adopt a very similar orientation as observed in docking pose and formed stable and strong interactions with SARS-CoV-2 M^pro^ (PDB ID:6LU7) with the key residues, Ser-144, His-163, and Glu-166. Based on our studies, the optimized cinanserin compounds are predicted to be viable drug candidates that can be pursued to develop COVID-19 therapeutics.

## Figures and Tables

**Table 1:** Residue Variation in the Active site of Coronavirus.

Residue Position	Subsite	Residue in SARS-CoV-2 M^pro^	Residue Variety
49	S2	Met	Leu, Ala, Met, Tyr, Ser, Phe, Asn, Thr
50	S2	Leu	Val, Thr, Asn, Gln, Lys, His, Ser, Arg, Met, Ala, Leu
190	S4	Thr	Thr, Val, Lys, Asn, Cys, Ile, Arg, Ser, Leu
191	S4	Ala	Val, Ile, Pro, Phe, His, Tyr, Ser, Met, Leu, Ala
193	S4	Val	Lys, Asn, Gln, Ile, Val, Ala, Leu, Met, Phe, Arg, His, Ser

**Table 2:** Docking scores of successfully docked zinc database coronavirus inhibitors from PDB structures.

Zinc Database Inhibitor	PDB File of Compound	Abbreviated Compound Name in PDB File	Docking Score −log_10_(Kd)
Z1587220559	5REC	T1J	5.92
Z271004858	5RF8	SFY	5.42
PCM-0102269	5RET	T47	4.69
Z1271660837	5RFB	K3S	3.80
PCM-0102575	5RFK	T7D	3.47

**Table 3:** Docking Scores of T47 and T1J derivatives with halogen substituents.

Compound	Highest Docking Score −log_10_(Kd)
**T1J G-series**	**5.92**
GM03	5.64
GN01	5.43
GN02	5.39
GM10	4.82
**T47 chair B-series**	**4.69**
BM26	4.68
BD11	4.66
BD23	4.43
BM24	4.17
BD22	4.25
BM25	4.08

Bolded compounds are the parent compounds and are shown for comparison.

**Table 4:** Docking scores for addition of aliphatic substituents to T47 and SFY compounds.

Compound	Highest Docking Score −log_10_(Kd)	Substituent Added	Compound	Highest Docking Score −log_10_(Kd)	*o, m, p*	Substituent Added
HS04	6.30	Butyl	SD12	7.62	*m*	Butyl
BD03	5.92		SD11	7.28	*m*	Propyl
HS03	5.68	Propyl	SD23	7.17	*m*	Neopentyl
BD02	5.41		SD15	7.16	*p*	Propyl
BD01	5.40		SD27	7.04	*m*	Butenyl
HS01	5.05	Methyl	SD28	6.79	*p*	Butenyl
HS02	4.95	Ethyl	SD07	6.66	*o*	Propyl
**T47**	**4.69**		SD08	6.53	*o*	Butyl
			SD24	6.45	*p*	Neopentyl
			SD16	6.35	*p*	Butyl
			SD14	6.20	*p*	Ethyl
			SD26	6.10	*o*	Butenyl
			SD09	6.10	*m*	Methyl
			SD22	6.07	*o*	Neopentyl
			SD10	5.95	*m*	Ethyl
			SD13	5.88	*p*	Methyl
			SD06	5.70	*o*	Ethyl
			SD05	5.51	*o*	Methyl
			**SFY**	**5.42**		

Bolded compounds are the parent compounds and are shown for comparison.

**Table 5:** Docking Scores for nitrogen-containing heterocycle derivatives of T47 and SFY.

Compound	Highest Docking Score −log_10_(Kd)	Compound	Highest Docking Score −log_10_(Kd)
DB04	6.88	SD29	7.72
DB09	6.76	SD25	7.19
DB11	6.77	SD19	6.83
DB03	6.65	SD31	6.68
DB12	6.33	SD18	6.21
DB02	6.32	SD20	5.46
DB10	6.22	**SFY**	**5.42**
DB08	5.97	SD30	5.42
DB05	5.53	SD17	5.40
DB06	5.39	SD32	4.69
**T47**	**4.69**	SD33	4.31

Bolded compounds are the parent compounds and are shown for comparison.

**Table 6:** Docking Scores of SFY and K3S derivatives having aliphatic rings.

Compound	Highest Docking Score −log_10_(Kd)	OMP	Substituent(s)	Compound	Highest Docking Score −log_10_(Kd)
SD34	11.02	M/P	Butyl/butyl	KT11	7.00
LEA4	10.94	O/P	Butyl/butyl	E010	6.97
LEA2	10.02	O/P	Propyl/propyl	KT10	6.36
KT04	9.92	P	Butyl	KT14	6.09
KTH1	9.82	O/P	Ethyl/butyl	KT12	5.72
KTH3	9.82		Butyl/cyclohexyl	EW10	5.52
SD35	9.72	M/P	Propyl/propyl	EW01	4.36
KTH2	9.64	O/P	Methyl/butyl	EW06	4.27
LEA3	9.54	O/P	Ethyl/ethyl	EW08	4.17
SD04	9.41	M	Butyl	**K3S**	**3.80**
SD03	9.33	M	Propyl	EW07	3.69
SD36	9.21	M/P	Ethyl/ethyl		
KT03	9.21	P	Propyl		
LM03	9.12	O	Butyl		
LEA1	8.81	O/P	Methyl/methyl		
L006	8.75	O	Propyl		
KT02	8.71	P	Ethyl		
SD37	8.62	M/P	Methyl/methyl		
SD02	8.52	M	Ethyl		
L005	8.10	O	Ethyl		
L001	8.26				
LM02	8.28				
KT01	7.91	P	Methyl		
SD01	7.83	M	Methyl		
SD21	7.74				
L004	7.60	O	Methyl		
**SFY**	**5.42**				

Bolded compounds are the parent compounds and are shown for comparison.

**Table 7:** Docking scores for T47, T1J, and T7D derivatives having hydrogen acceptors and donor atoms.

Compound	Highest Docking Score −log_10_(Kd)	Compound	Highest Docking Score −log_10_(Kd)
KF08	8.80	JN16	8.16
KF03	8.07	JMH5	7.42
KF04	7.14	JN14	6.00
KF07	6.90	JCN6	5.82
KF01	6.77	JCN8	5.79
KF02	6.66	JN12	5.61
GN06	6.41	JN10	5.28
**T1J**	**5.92**	**T7DM**	**3.77**
GN07	5.67	**T7D**	**3.47**
GM09	5.61		
KF05	5.52		
EW13	7.33	HS06	7.85
EW14	7.32	HS05	7.03
EW11	6.99	**T47**	**4.69**

Bolded compounds are the parent compounds and are shown for comparison.

**Table 8:** Docking scores, LogP, and LogS values for optimized compounds and parent zinc database inhibitors.

Compound	Highest Docking Score −log_10_(Kd)	LogP	LogS
FL30	13.98	1.62	−4.27
FL20	13.17	1.44	−3.76
FL29	12.98	4.39	−5.51
FL28	12.98	1.62	−4.01
FL23	12.96	4.25	−5.38
FL26	12.67	4.35	−5.56
FL16	12.41	0.79	−3.23
FL22	12.32	1.43	−3.72
FL24	12.30	4.23	−5.32
KBH1	11.31	1.96	−5.48
FL05	11.11	0.45	−2.67
FL18	11.09	1.00	−3.56
FL14	10.83	1.15	−3.65
FL09	10.62	0.63	−2.82
FL04	10.60	0.45	−2.64
FL21	10.50	2.00	−3.58
KB01	10.46	0.90	−4.46
FL27	10.44	0.58	−2.87
FL31	10.27	1.39	−3.79
FL15	10.10	1.13	−3.65
FL06	10.05	0.86	−2.91
FL08	9.92	0.11	−2.38
FL07	9.82	0.14	−2.38
FL25	9.42	0.29	−2.84
FL03	9.26	1.26	−3.36
**T1J**	**5.92**	**2.83**	**−3.16**
**SFY**	**5.42**	**0.84**	**−3.03**
**T47**	**4.69**	**1.73**	**−2.02**
**K3S**	**3.80**	**−0.27**	**−1.43**
**T7D**	**3.47**	**1.73**	**−2.02**

Bolded compounds are the parent compounds and are shown for comparison.

**Table 9:** Docking scores, predicted LogP, predicted LogS, topological polar surface area (TPSA), and protease bioactivity values of successfully docked cinanserin and optimized cinanserin compounds.

Inhibitor Name	Highest Docking Score −log_10_(Kd)	LogP	LogS	MW	Topological Polar Surface Area	Protease Bioactivity Score
CM06	10.60	3.61	−5.74	402.61	80.91	0.51
CM07	9.12	3.75	−6.03	410.59	93.17	0.11
CM02	8.98	5.23	−6.12	416.63	75.35	0.46
CO08	8.95	5.51	−6.54	404.63	55.12	0.49
CM05	8.42	4.39	−6.13	417.25	58.36	0.56
CM01	8.29	2.66	−4.58	407.67	58.36	0.76
CO09	8.19	5.51	−6.55	400.63	55.12	0.47
CO03	8.15	3.41	−5.06	367.60	58.36	0.60
CO04	8.05	2.53	−4.85	361.56	58.36	0.46
CO10	7.28	5.84	−6.63	394.58	55.12	0.21
**Cinanserin**	**6.01**	**4.27**	**−5.24**	**340.49**	**32.34**	**−0.10**

Bolded compounds are the parent compounds and are shown for comparison.

## Supplementary Materials

The following are available online at https://www.mdpi.com/1999-4915/12/9/942/s1, Table S1: Docking scores of 67 zinc database inhibitors in 6LU7 and 5R7Z actives sites. Supporting Information, Figure S1: The Root Mean Square Fluctuation (RMSF) plot based on C-α atoms of SARS-CoV-2 Mpro (PDB ID: 6LU7) complex with (A) CM02; (B) CM06; and (C) CM07.; and Supporting Information, Figure S2: Analysis of (A) molecular interactions; and (B) type of contacts (2D interaction contour map with key protein residues) for CM07 with SARS-Cov-2 M^pro^ (PDB:6LU7) after MD simulation.
