# Supplementary material for: Optimization Rules for SARS-CoV-2 Mpro Antivirals: Ensemble Docking and Exploration of the Coronavirus Protease Active Site
Source: Viruses. 2020 Aug 26;12(9):942. doi: 10.3390/v12090942 (PMC7552026; doi:10.3390/v12090942)
Supplement: Supplementary file 1 [file viruses-12-00942-s001.pdf]

Supplementary Table 1: Docking scores for Zinc database inhibitors in				
PDB File	5R7Z –$\log_{10}$(Kd)	5R7Z-CS –$\log_{10}$(Kd)	6LU7-CS –$\log_{10}$(Kd)	Compound
6W63	4.383	7.1804	7.0446	X77
5REC	4.77	5.9197	5.7341	Z1587220559
5RF8	4.38	5.422	5.4206	Z271004858
5RF7	5.42	5.371	5.3587	Z316425948_minor
5R84	3.214	4.8417	5.6658	Z1262246195
5RE9	4.836	4.5812	5.5103	Z2856434836
5R7Z	4.48	4.5759	5.2858	Z1220452176
5RFD	2.784	4.5417	4.4952	Z126932614
5REE	5	4.4794	4.6783	Z2217052426
5REX	3.374	4.4731	4.4132	PCM-0102287
5REY	3.259	4.3204	3.7805	PCM-0102911
5RE8	4.235	4.3139	4.404	Z2737076969
5REL	3.55	4.3093	4.4488	PCM-0102340
5REN	2.292	4.2897	4.5277	PCM-0102425
5RFP	3.88	4.2724	3.1721	PCM-0102190
5RE6	1.822	4.2561	5.7345	Z54571979
5RF0	2.903	4.2519	5.2252	POB0073
5RET	4.68	4.1565	4.3177	PCM-0102269
5RFL	3.258	4.1123	4.3131	PCM-0102389
5R82	2.609	4.0704	4.8051	Z219104216
5RFW	3.085	3.9684	3.6825	PCM-0102243
5RFR	2.775	3.9552	3.9275	PCM-0102169
5RF6	1.58	3.9388	4.8775	Z1348371854
5RFH	3.039	3.9254	3.193	PCM-0102277
5RFS	3.014	3.8521	4.1247	PCM-0102739
5RFB	3.74	3.8016	3.6927	Z1271660837
5RFJ	2.199	3.7551	3.8153	PCM-0103067
5REW	2.625	3.7105	3.3532	PCM-0102275
5RE7	3.26	3.7039	4.4737	Z30932204
5REG	3.022	3.6734	5.6769	Z1545313172
5REZ	2.771	3.5535	3.7781	POB0129
5REF	3.651	3.4622	2.6277	Z24758179
5RF4	2.149	3.4302	4.7708	Z1741982125
5R81	1.774	3.4155	3.2934	Z1367324110
5REO	2.947	3.4091	3.1781	PCM-0102578
5RF9	2.635	3.4047	3.0218	Z217038356
5RFA	2.496	3.2041	4.1453	Z2643472210
5RFX	2.761	3.1372	2.8674	PCM-0102254
5R7Y	2.98	3.0971	4.025	Z45617795
5RFQ	2.635	3.0684	4.1089	PCM-0102179
5RFY	3.115	3.0024	2.409	PCM-0102974

5RE4	2.424	2.9398	4.5754	Z1129283193
5RFT	2.193	2.8883	2.5828	PCM-0102432
5RFO	2.108	2.8086	2.4942	PCM-0102972
5RES	2.499	2.7857	2.6313	PCM-0102281
5REI	2.553	2.779	3.2856	Z2856434856
5REK	2.314	2.7101	2.9503	PCM-0102327
5RFZ	1.849	2.6799	4.1185	PCM-0102274
5RF3	1.746	2.6759	3.7383	Z1741970824
5REM	2.096	2.6232	1.3751	PCM-0103016
5REV	2.5	2.5145	4.0126	PCM-0103072
5RFI	2.862	2.5014	2.7716	PCM-0102353
5RED	2.412	2.4959	2.5502	Z2856434865
5REU	2.325	2.445	2.1136	PCM-0102395
5RFK	3.47	2.357	2.4723	PCM-0102575
5R80	1.86	2.2846	2.7237	Z18197050
5REA	2.102	2.2754	3.1157	Z31432226
5RFV	2.193	2.2753	2.2122	PCM-0102306
5RF1	1.195	2.2556	2.3627	NCL-00023830
5RF5	2.168	2.2053	2.584	Z3241250482
5REJ	2.421	2.1995	1.5755	PCM-0102241
5REP	2.397	2.1369	2.0448	PCM-0102201
5RG0	1.541	2.1141	0.9612	PCM-0102535
5RER	2.454	2.0985	2.4968	PCM-0102615
5RFU	1.793	1.5661	1.4663	PCM-0102121
5RFF	1.839	1.3296	2.2905	PCM-0102704

A

CM-02

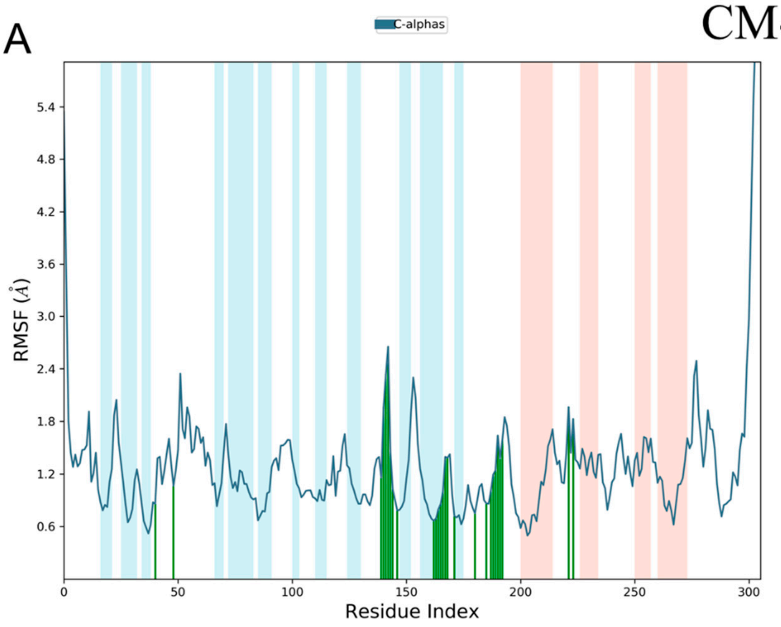

B

CM-06

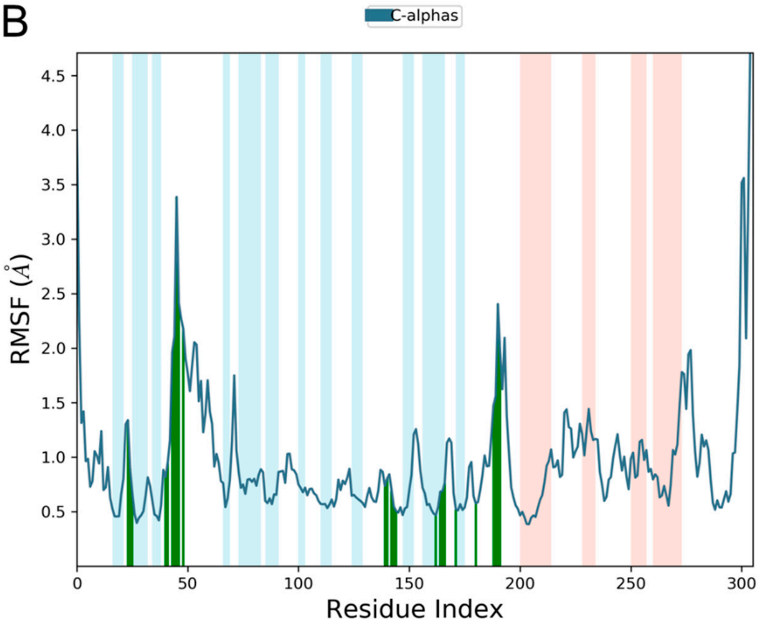

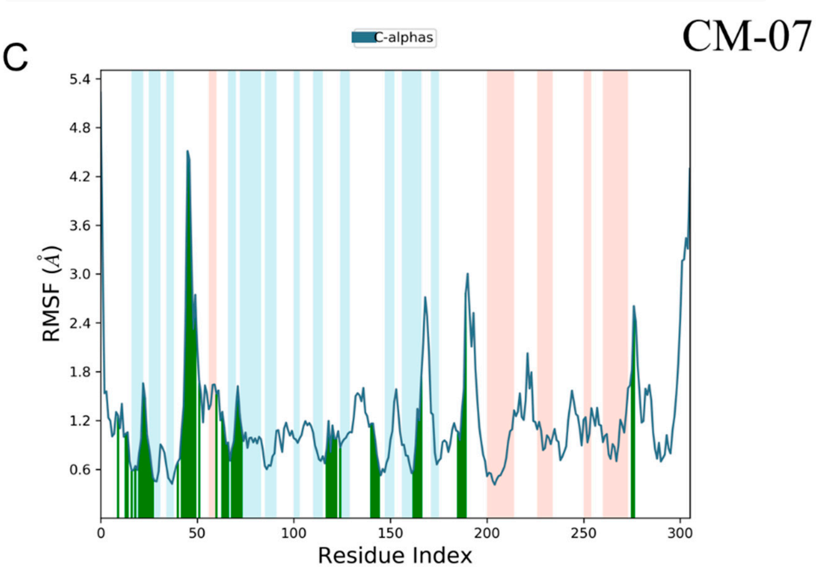

Figure S1. The Root Mean Square Fluctuation (RMSF) plot based on C-α atoms of SARS-CoV-2 Mpro (PDB ID: 6LU7) complex with (A) CM02; (B) CM06; and (C) CM07. Protein residues that interact with above compounds are marked with green vertical bars.

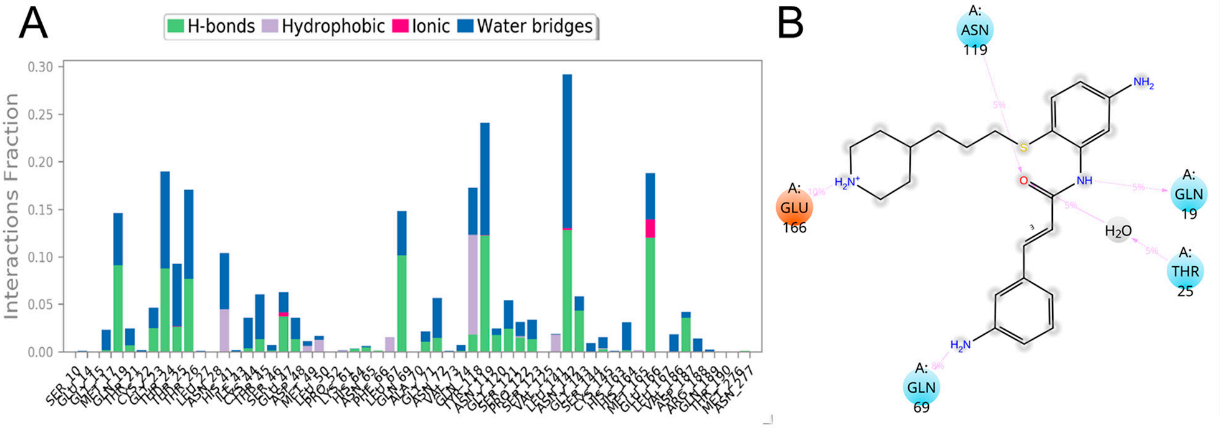

Figure S2. Analysis of (**a**) molecular interactions; and (b) type of contacts (2D interaction contour map with the key protein residues) for CM07 with SARS-CoV-2 M^{pro} (PDB ID: 6LU7) after MD simulation.
